# The Agr Quorum Sensing System Represses Persister Formation through Regulation of Phenol Soluble Modulins in *Staphylococcus aureus*

**DOI:** 10.3389/fmicb.2017.02189

**Published:** 2017-11-07

**Authors:** Tao Xu, Xu-Yang Wang, Peng Cui, Yu-Meng Zhang, Wen-Hong Zhang, Ying Zhang

**Affiliations:** ^1^Key Laboratory of Medical Molecular Virology, Huashan Hospital, Shanghai Medical College of Fudan University, Shanghai, China; ^2^Department of Molecular Microbiology and Immunology, Bloomberg School of Public Health, Johns Hopkins University, Baltimore, MD, United States

**Keywords:** *Staphylococcus aureus*, Agr, antibiotic, persister formation, sialic metabolism

## Abstract

The opportunistic pathogen *Staphylococcus aureus* has become an increasing threat to public health. While the Agr quorum sensing (QS) system is a master regulator of *S. aureus* virulence, its dysfunction has been frequently reported to promote bacteremia and mortality in clinical infections. Here we show that the Agr system is involved in persister formation in *S. aureus*. Mutation of either *agrCA* or *agrD* but not *RNAIII* resulted in increased persister formation of stationary phase cultures. RNA-seq analysis showed that in stationary phase AgrCA/AgrD and RNAIII mutants showed consistent up-regulation of virulence associated genes (*lip* and *splE*, etc.) and down-regulation of metabolism genes (*bioA* and *nanK*, etc.). Meanwhile, though knockout of *agrCA* or *agrD* strongly repressed expression of phenol soluble modulin encoding genes *psmα1-4*, *psm*β*1-2* and phenol soluble modulins (PSM) transporter encoding genes in the *pmt* operon, mutation of RNAIII enhanced expression of the genes. We further found that knockout of *psmα1-4* or *psm*β*1-2* augmented persister formation and that co-overexpression of PSMαs and PSMβs reversed the effects of AgrCA mutation on persister formation. We also detected the effects on persister formation by mutations of metabolism genes (*arcA*, *hutU*, *narG*, nanK, etc.) that are potentially regulated by Agr system. It was found that deletion of the ManNAc kinase encoding gene *nanK* decreased persister formation. Taken together, these results shed new light on the PSM dependent regulatory role of Agr system in persister formation and may have implications for clinical treatment of MRSA persistent infections.

## Introduction

*Staphylococcus aureus*, as a leading bacterial agent of a series of hospital- and community- acquired infections, has been listed as a pathogen with high priority for research and development of new antibiotics (World Health Organization, 2017). Fail of eradication of *S. aureus* in clinical treatments is linked to persister formation. Persisters, first observed in staphylococci in Hobby et al. (1942), are defined as a subpopulation of bacterial cells that, without undergoing genetic changes, survive the effects of high concentration of bactericidal antibiotics (Bigger, 1944). Persisters have been associated with persistent infections, presenting severe threats to patients with bacterial infections (Lewis, 2007; Fauvart et al., 2011; Zhang, 2014).

The last decade has seen much research on the mechanisms of persister formation, most of which was derived from *Escherichia coli*. Genes involved in toxin–antitoxin modules, *trans*-translation, energy production, stringent response and efflux have been found to be involved in persister formation by inducing dormancy (Dorr et al., 2009; Kim et al., 2009; Ma et al., 2010; Li et al., 2013; Pu et al., 2016). The most in-depth study so far, though not necessarily the most important, is on HipAB module in *E. coli.* Mutated HipA augments persister formation by phosphorylation of GltX, which in turn raises concentration of uncharged tRNA^Glu^, induces production of (p)ppGpp and polyP, and eventually promotes persister formation with increased production of toxins including HipA, RelE, MazF, and YafQ (Korch et al., 2003; Maisonneuve et al., 2013; Germain et al., 2015; Mitchell et al., 2016). A recent study revealed a similar mechanism that a toxin TacT promotes persister formation via acetylation of tRNA in *Salmonella enterica* serovar Typhimurium (Cheverton et al., 2016). Research on persister formation has also been performed in other bacteria, including *Pseudomonas aeruginosa*, *Streptococcus mutans* and *Staphylococcus aureus* (Singh et al., 2009; Mulcahy et al., 2010; Leung and Lévesque, 2012).

Previous studies showed that the characteristics and mechanisms of persisters in *S. aureus* are different from those in *E. coli*. The higher portion of persisters in stationary phase and clinical impact of Staphylococcus persisters has drawn increasing attention recently (Fauvart et al., 2011; Lechner et al., 2012). It takes more time to show distinct killing of *S. aureus* cells, and an iconic feature of *E. coli* persisters, called biphasic killing curve, is often absent in treatment of stationary phase *S. aureus* cultures with antibiotics (Lechner et al., 2012; Conlon et al., 2013; Xu et al., 2016). Multiple genes have been reported to be associated with Staphylococcus persister formation (Mechler et al., 2015; Wenjie et al., 2015; Yee et al., 2015; Xu et al., 2016). In addition, L-forms that survive high concentration of antibiotics with cell wall deficiency are believed to be a special kind of persisters (Han et al., 2014). It is intriguing that few of the homologous genes of many *E. coli* persister genes affect persister formation in *S. aureus*. A typical case is that no mutation of known TA modules in *S. aureus* affects persister levels (Conlon et al., 2016). In addition, the ppGpp molecule is not as important in *S. aureus* persister formation as in *E. coli* (Geiger et al., 2010; Corrigan et al., 2016). Nonetheless, it is worth noting that besides specific genes, decreased ATP level during entry into stationary phase is found to be a key reason of persister increase in both *E. coli* and *S. aureus* (Conlon et al., 2016; Shan et al., 2017).

Besides the difference of persister genes between *E. coli* and *S. aureus*, the regulatory mechanism of *S. aureus* persister formation also remains elusive. Persister formation has been defined as an outcome of stochastic induction of toxin-antitoxin activity and other persister genes influenced by the level of p(ppGpp) which varies stochastically in different cells during growth (Maisonneuve et al., 2013; Germain et al., 2015). However, some key aspects of stochastic theory are unclear, especially the initiation of persister formation. QS, for its manner of regulation, is a candidate for this kind of regulation. In *P. aeruginosa* and *S. mutans*, regulation of QS has been reported (Moker et al., 2010; ?). However, whether the QS system is involved in *S. aureus* persister formation needs to be determined.

There are two known QS systems in *S. aureus*, LuxS and Agr. Agr is a master regulator of virulence, activated by an autoinducing peptide (AIP) that is encoded by *agrD* and modified and exported by AgrB (Ji et al., 1995; Morfeldt et al., 1995). Extracellular AIP molecules are processed by a TCS consisting of the histidine kinase AgrC and the response regulator AgrA (Lina et al., 1998). While most of downstream virulence genes are regulated through RNAIII, AgrA directly regulates several metabolic pathways including carbohydrate and amino acid metabolism (Dunman et al., 2001; Ziebandt et al., 2004; Queck et al., 2008). Also, Agr particularly controls expression of three specific group of virulence factors named PSMs (Wang et al., 2007; Peschel and Otto, 2013), which are transported by the Pmt system (Chatterjee et al., 2013). Joo et al. (2016) has recently reported that PSMs bind PmtR, the transcription repressor of the Pmt system, and hence promote expression of PSM transporters, showing that PSMs not only function as toxins but act as signals to regulate gene expression of *S. aureus*.

The correlation between persister formation and persistent infection is drawing increasing attention. Multiple observations of persisters in clinical infections have been reported in *E. coli*, *S. aureus*, *P. aeruginosa*, and *Mycobacterium tuberculosis* (Dhar and McKinney, 2007; Zhang et al., 2012; Cohen et al., 2013). High expression of HipA promotes persister formation and thus cause multidrug tolerance in urinary tract infections by *E. coli* (Schumacher et al., 2015). Despite clinical observations, a recent study showed that awakened *S. aureus* persisters are able to initiate infections and that *S. aureus* persisters, whether awake or not, possess advantages escaping from engulfment of macrophages (Mina and Marques, 2016), supporting the notion that persisters are important in initiating and establishing infections. Hence, understanding the mechanisms of *S. aureus* persister formation is critical for development of new strategies against the dangerous pathogen.

Here we report that Agr is a repressor of persister formation in stationary phase *S. aureus*. The modulation of persister formation by Agr is not RNAIII dependent, but relies on regulation of PSM expression. The findings provide new insights into the pathogenesis of *S. aureus* and clinical treatment of *S. aureus* infections.

## Materials and Methods

### Strains, Reagents, and Growth Conditions

*Staphylococcus aureus* MRSA strain USA500 (Diep et al., 2006) as well as MSSA strains HG003 (Herbert et al., 2010) and Newman (Baba et al., 2008) were used as wild type strains. *E. coli* DC10B (Monk et al., 2012) was used for shuttle plasmid construction (**Table 1**). All manipulations of the strains were carried out in biosafety level 2 labs.

**Table 1:** Strains and plasmids used in this study.

Strain/plasmid	Specification	Reference
**Strains**		
DC10B	A DC10B derivate for plasmid amplification	Monk et al., 2012
USA500	MRSA strain from a clinical sample	Diep et al., 2006
Newman	An MSSA strain from a clinical sample	Baba et al., 2008
HG003	An MSSA strain, derivate from NCTC8325	Herbert et al., 2010
ΔAgrCA	The *agrCA* mutant of USA500	This study
NewmanΔAgrCA	The *agrCA* mutant of Newman	This study
HG003ΔAgrCA	The *agrCA* mutant of HG003	This study
ΔAgrD	The *agrD* mutant of USA500	This study
ΔRNAIII	The *RNAIII* mutant of USA500	This study
ΔluxS	The *luxS* mutant of USA500	This study
ΔArlRS	The *arlRS* mutant of USA500	This study
ΔGraRS	The *graRS* mutant of USA500	This study
ΔNanK	The *nanK* mutant of USA500	This study
ΔAgrCAΔNanK	The *agrCA* mutant of USA500 with additional knockout of *nanK*	This study
ΔHutU	The *hutU* mutant of USA500	This study
ΔNarG	The *narG* mutant of USA500	This study
ΔArcA	The *arcA* mutant of Newman	This study
ΔArcR	The *arcR* mutant of Newman	This study
ΔMtlD	The *mtlD* mutant of Newman	This study
ΔImrP	The *imrP* mutant of Newman	This study
ΔRhbC	The *rhbC* mutant of Newman	This study
ΔIpdC	The *ipdC* mutant of Newman	This study
**Plasmids**		
pRAB11	ATc inducible shuttle plasmid, Cm^R^, Amp^R^	Helle et al., 2011
pMX10	A pKOR1 derivate for gene knockout, Cm^R^, Amp^R^	This study
pRB473	A shuttle plasmid for complementary construction, Cm^R^, Amp^R^	Bruckner et al., 1993
pRBagrCA	Complementary plasmid for AgrCA	This study
pRBagrD	Complementary plasmid for AgrD	This study
pRBnanK	Complementary plasmid for NanK	This study
pRABpsmα	Overexpression plasmid for *psmα1-4*	This study
pRABpsmβ	Overexpression plasmid for *psm*β*1-2*	This study
pRABpsmαβ	Overexpression plasmid for *psmα1-4* and *psm*β*1-2*	This study

*Cm^*R*^, chloramphenicol resistance; Amp^*R*^, ampicillin resistance*.

Lysogeny broth (LB) was used for cultivation of *E. coli* strains. TSB (Tryptic soy broth, Oxoid) were used for *S. aureus* cultivation. Anhydrotetracycline (ATc) (Sigma) was used for induction of *secY* antisense RNA during screening of mutants. During cultivation of strains that carry antibiotic resistance genes, antibiotics were added to medium at the following concentrations: chloramphenicol, 10 μg/μl; ampicillin, 100 μg/μl.

All strains were cultured at 37°C with shaking at 180 rpm unless otherwise specified. Growth curves were determined by measuring the optical density (OD) values at 600 nm every hour.

### Gene Knockout, Complementation, and Overexpression

Plasmid pMX10 (Xu et al., 2016), a derivate of pKOR1 (Bae and Schneewind, 2006), was used for gene knockout in *S. aureus*. Plasmid pRB473 (Bruckner et al., 1993) was used for complementation of gene mutations. Plasmid pRAB11 (Helle et al., 2011) was used for inducible expression of target genes. Q5 DNA polymerase (NEB) was used for all PCR experiments and restriction enzymes (NEB) for construction of recombinant plasmids in this study. Additional information of plasmids used in this study can be found in **Table 1**.

All sequences of primers are listed in Supplementary Table S1. To construct knockout mutants, upstream and downstream fragments of each gene were amplified with primers uf+ur and df+dr, respectively, using genome DNA of HG003, USA500 or Newman as templates. The two fragments were then used as templates to amplify a fusion fragment, which was ligated into pMX10 with T4 DNA ligase (Thermo Fisher Scientific) after digestion with corresponding restriction enzymes (Thermo Fisher Scientific). The recombinant plasmids were purified from DC10B and introduced into *S. aureus* via electro transformation. Selection of mutants were carried out following the protocol previously published (Bae and Schneewind, 2006).

To construct complementary strains, genes with their own promoters were amplified from USA500 genomic DNA. Since the coding sequences of *agrCA* and *agrD* are not directly attached to that P2 promoter of *agr* operon, we fused the P2 promoter sequence and the coding sequence of *agrCA* or *agrD*, similar with construction of knockout plasmids described above. The PCR products were digested with corresponding restriction enzymes and ligated into pRB473. To construct plasmids for inducible overexpression, the full sequence of *psmα1-4* or *psm*β*1-2* were amplified and inserted into pRAB11. For co-expression of PSMαs and PSMβs, the coding sequences of *psmα1-4* and *psm*β*1-2* were fused and cloned into pRAB11. The resultant plasmids were transformed to each mutant strain via electro transformation.

### Susceptibility Testing

The MIC of each antibiotic was determined in TSB medium by the conventional broth micro dilution technique. The experiments were executed in triplicate according to the protocol previously published (Andrews, 2001), following the CLSI guidelines. The MIC was defined as the lowest antibiotic concentration that inhibited visible bacterial growth after 24 h of incubation at 37°C.

### Persister Assay

To obtain exponential cultures, overnight *S. aureus* strains were inoculated by 1:100 into TSB and cultured for about 1.5 h to an OD600 of 0.5–0.6. Stationary phase cultures were grown to ∼14 h. For complementary strains, the cultures were grown in TSB with 10 μg/μl chloramphenicol. For strains for inducible overexpression, 125 ng/μl ATc was added to each sample at 6 h. After washing twice with PBS, cultures were allotted in 14 μl tubes and challenged with one of the four antibiotics: levofloxacin at 50 μg/μl, gentamicin at 50 μg/μl, oxacillin at 5 μg/μl, and vancomycin at 50 μg/μl. Serial dilutions were performed and 100 μl aliquots were spotted on TSA plates for colony-forming unit (CFU) counting at different time points. Results were obtained from three biological duplicates and significance was assessed with *t*-test.

### RNA Isolation, mRNA Enrichment and Sequencing

*Staphylococcus aureus* strain USA500, Δ*agrD*, Δ*agrCA*, and Δ*RNAIII* were incubated for 14 h and harvested. Total RNA was purified following protocol previous reported (Atshan et al., 2012). Briefly, cultures were centrifuged, resuspended in 100 μl diethylpyrocarbonate (DEPC) H_2_O. Each aliquot was added with 100 μl phenol/chloroform (1:1), incubated at 70°C for 30 min and centrifuged. RNA was purified from the supernatant of each sample using RNeasy Mini Kit (Qiagen), following protocol provided.

The Bioanalyzer 2100 RNA-6000 Nano Kit (Agilent Technologies) was used to detect the quality of total RNA. The Ribo-Zero^TM^ Gold Kit (Illumina) was used to remove 16S and 23S rRNAs. The cDNA libraries with cDNA length ranging from 150 to 250 bp were generated from the mRNA samples using the TruSeq Illumina Kit (Illumina), following instructions from the manufacturer.

RNA-seq was performed by Shanghai Biotechnology Corporation (Shanghai, China) with HiSeq2500 Ultra-High-Throughput Sequencing System (Illumina). The total numbers of reads were assessed and quantified with the Cufflinks suite of tools. The transcript sequencing data were submitted to the NCBI Sequence Read Archive and deposited under the accession number SRR5277864.

### Quantitative Real-Time PCR

For qRT-PCR, RNA samples were extracted from *S. aureus* strains as described above. Reverse transcription was carried out using the cDNA Synthesis Kit (Bio-Rad, United States). The qRT-PCR experiments were performed using SYBR Green PCR Kit (Takara) on Applied Biosystems 7500 Real-Time PCR System (Thermo Fisher Scientific). The sequences of primers for detection of target genes and primers for endogenous control gene *rrs1* (16S RNA) are listed in Supplementary Table S1. The 2^-ΔΔC_T_^ method (Livak and Schmittgen, 2001) was used for analysis of relative gene expression data that were obtained from three independent experiments.

### L-Form Assay

L-form test was performed following protocol previously published (Han et al., 2014). Briefly, L-form induction medium (LIM) was prepared with brain heart infusion (BHI) supplemented with 1% agar, 10% fetal bovine serum (Gibco, United States), 3.5% sodium chloride, 20% sucrose, 0.125% magnesium sulfate, and 600 μg (1000 units)/μl of Penicillin G (Sigma). *S. aureus* Newman strain was grown overnight to stationary phase. Undiluted cultures were spotted onto LIM and incubated at 33°C for 7–10 days. The L-form colonies were detected by inverted microscope (Nikon).

### Measurement of Intracellular ATP Level

Stationary phase cultures of *S. aureus* strains were mixed with BacTiter-Glo^TM^ reagent from the BacTiter-Glo^TM^ Microbial Cell Viability Assay (Promega, United States) by 1:1. The luminescence was detected with an FB12 luminometer (Berthold). Data was neutralized according to CFU counting of each culture.

### Statistics

The significance of experimental differences in persister assay and intracellular ATP assay was evaluated with two-tailed unpaired *t*-test (two groups).

## Results

### Agr System Regulates Persister Formation

**FIGURE 1:**
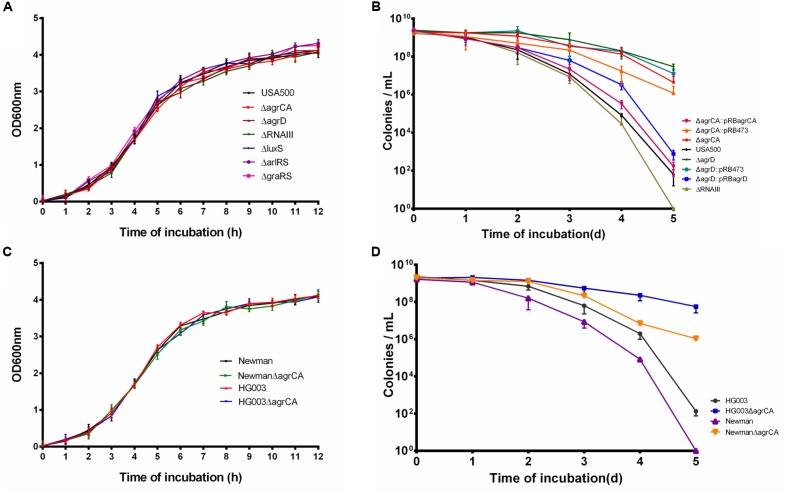
Agr system regulates *S. aureus* persiser levels. **(A,C)** Growth curve of different knockout strains compared with their parent strains. **(B,D)** Stationary phase cultures of knockout strains and the parent strains were treated with 50 μg/μl levofloxacin. The limit of detection was 100 CFU/μl throughout all killing experiments. Results are representative of three independent experiments.

We constructed mutants via homologous recombination for Agr and LuxS QS systems, as well as two TCSs ArlRS and GraRS involved in virulence regulation. Considering the complexity of Agr system, mutation of the AIP coding gene *agrD* and the Agr controlled regulatory RNA molecule gene *RNAIII* were also constructed (**Table 2**). The growth of each mutant strain of USA500 was measured. As shown in **Figure 1A**, none of the knockout mutants affected bacterial growth, indicating these genes are not required for normal bacterial growth in log phase.

**Table 2:** Genes detected for persister formation in this study.

Gene ID	Gene	Function	Parent	Persister-	Persister-
	name		strain	exponential	stationary
				culture	culture
**Regulators**					
*USA300HOU_2034, USA300HOU_2035*	*agrCA*	Two-component system	USA500	–	↑
*NWMN_1945, NWMN_1946*	*agrCA*	Two-component system	Newman	–	↑
*SAOUHSC_02264, SAOUHSC_02265*	*agrCA*	Two-component system	HG003	–	↑
*USA300HOU_2033*	*agrD*	Signal peptide precursor	USA500	–	↑
*USA300HOU_nc0020*	*RNAIII*	Regulatory RNA, includes *hld coding sequence*	USA500	–	↓
*USA300HOU_2123*	*luxS*	Quorum sensing	USA500	–	–
**Transcription regulators**					
*USA300HOU_0680*, *USA300HOU_0681*	*graRS*	Two-component system	USA500	–	–
*USA300HOU_1350*, *USA300HOU_1349*	*arlRS*	Two-component system	USA500	–	–
**Metabolism genes**					
*USA300HOU_0336*	*nanK*	ManNAc kinase	USA500	–	↓
*USA300HOU_2311*	*hutU*	Urocanate hydratase	USA500	–	–
*USA300HOU_2379*	*narG*	Nitrate reductase	USA500	–	–
*NWMN_2534*	*arcA*	Arginine deiminase	Newman	–	–
*NWMN_2530*	*arcR*	Arginine metabolism regulator	Newman	–	–
*NWMN_2060*	*mtlD*	mannitol-1-phosphate 5-dehydrogenase	Newman	–	–
*NWMN_0122*	*imrP*	Transporter	Newman	–	–
*NWMN_2082*	*rhbC*	Sialic synthase	Newman	–	–
*NWMN_0132*	*ipdC*	Indole-3-pyruvate decarboxylase	Newman	–	

*↑, increase; ↓, decrease; –, no significant difference*.

To address the role of the above genes in persister formation, several antibiotics were used to treat exponential phase cultures of USA500 and the mutant strains. As a result, none of the mutants affected persister formation to any of the antibiotics (**Supplementary Figures S1A–D**). Stationary cultures of MRSA can be extremely difficult to kill by multiple antibiotics, even after 5-day treatment at high concentrations of antibiotics (Lechner et al., 2012; Conlon et al., 2013). Based on our previous observation that levofloxacin can cause prominent killing of *S. aureus* USA500 strain (Xu et al., 2016), we used levofloxacin in persister assays of stationary phase cultures in this study. Among the strains, USA500 Δ*agrCA* or USA500 Δ*agrD* showed significant increase of persister formation to levofloxacin in stationary phase while knockout of *RNAIII* slightly reduced persister formation. At the 5th day of treatment, the CFU count of USA500 Δ*agrCA* or USA500 Δ*agrD* dropped ∼1 log_10_ compared to that of the 1st day, while the CFU count of USA500 ΔRNAIII (∼10 CFU/μl) was even lower than that of the control strain USA500 (∼10^2^ CFU/μl). The impacts of knockout of *agrCA* or *agrD* were reversed by complementation of each gene (**Figure 1B**). Mutation in *luxS*, *arlRS*, or *graRS* showed no apparent impact on persister formation of stationary phase cultures (**Supplementary Figure S1E**).

To investigate whether the impact on persister formation by Agr system is ubiquitous in *S. aureus*, we constructed *agrCA* mutants in two methicillin sensitive *S. aureus* (MSSA) strains Newman and HG003. While none of the knockout mutations affected growth (**Figure 1C**), both mutant strains showed augmented persister formation in stationary phase, but only to levofloxacin (**Figure 1D**).

### RNA-seq Analysis of Stationary Phase Regulation by Agr System

The Agr system functions by regulating expression of its target genes, either by direct regulation with AgrA or by indirect regulation through RNAIII (George and Muir, 2007; Queck et al., 2008). As we have shown, the difference in bacterial survival between Δ*agrCA* and Δ*RNAIII* mutants indicates that the regulation of persister formation by Agr system is RNAIII independent. According to the study by Queck et al. (2008) with RNA-seq analysis of 4-h cultures of *S. aureus* MW2 strain and its mutants of *agr* or *RNAIII*, Agr regulated genes that are RNAIII independent mainly consist of metabolism associated genes and PSM genes (Queck et al., 2008). Since the effects of AgrCA mutation were observed only in stationary phase, we performed RNA-seq with stationary phase cultures of USA500 and its mutants of *agrCA*, *agrD*, and *RNAIII*, to further investigate the RNAIII independent regulatory mechanisms of Agr system on persister formation.

**FIGURE 2:**
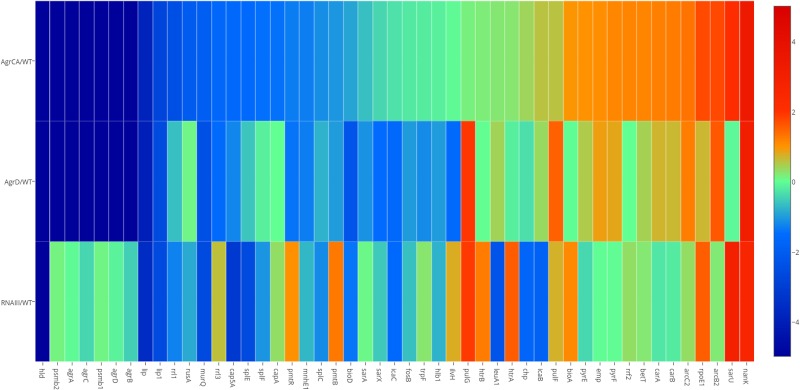
Transcriptional changes caused by mutation of *agrCA*, *agrD* and *RNAIII* in USA500. Based on results of RNA-seq, comparison (*p* < 0.05) of transcriptional levels of each strain stress with that of USA500 are shown in columns. Genes up regulated in each deletion strain are represented in red, whilst genes down regulated in green. The enriched GO terms in the resulting clusters are shown. The map was painted with MEV software. A3, Δ*agrCA*; D4, Δ*agrD*; R5, Δ*RNAIII*; WT, wild type.

As shown in the Heatmap (**Figure 2**), the three mutants caused significant changes to transcription of different genes, compared with the parent strain USA500. In the *agrCA* and *agrD* mutants, expression of metabolism genes including *bioA*, *pyrF*, and *nanK* were upregulated while several pathogenicity associated genes (*lip*, *lip1*, *splE*, and *splF*, etc.) were down regulated; meanwhile, the expression of PSM associated genes *hld*, *psmβ1*, *psmβ2*, *pmtR*, and *pmtB* were strongly repressed. Comparison of USA500ΔRNAIII with USA500ΔagrCA strain showed consistent down-regulation of virulence genes (*lip*, *lip1*, *splE*, and *splF*, etc.) and up-regulation of metabolism genes (*bioA*, *rpoE1*, and *nanK*, etc.), while unlike in USA500ΔagrCA, expression of *psmβ1*, *psmβ2*, *pmtR*, and *pmtB* in USA500ΔRNAIII were up regulated (Supplementary Tables S2, S4). It is worth mentioning that though the impact on gene expression by AgrD deletion was similar as that of AgrCA deletion, expression of several genes including *pulG* and *sarU* were regulated by AgrCA and AgrD differently (Supplementary Tables S2, S3).

**FIGURE 3:**
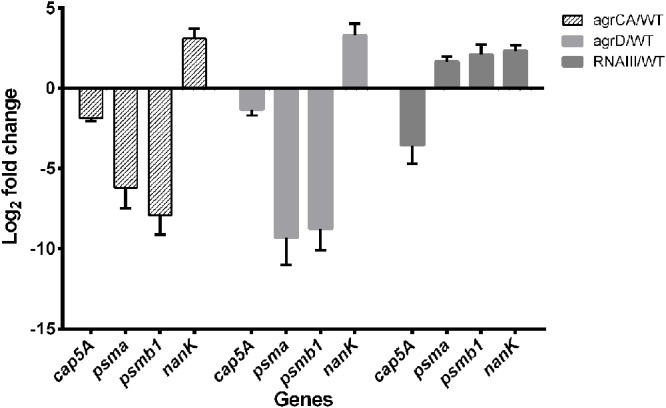
Validation of RNA-seq by quantitative real-time PCR. Relative mRNA levels of transcripts from to USA500 and *agrCA*, *agrD* as well as *RNAIII* mutant strains mutant grown to 12–14 were determined. RNA was obtained from the same samples for RNA-seq. Bars show the fold change of each mutant vs. USA500 and error bars indicate standard deviations calculated with the 2^-ΔΔC_t_^ method based on three independent experiments.

The RNA-seq results were validated by detecting transcription levels of several genes with Quantitative Real-time PCR. All samples showed similar fold change with those from RNA-seq results (**Figure 2**). The expression levels of the co-transcribed *psmα1-4* genes did not show up in RNA-seq data analysis of either strain, probably because the sizes of the four genes are too small to be included in the cDNA library. We detected mRNA level of *psmα* genes using primers for amplification of an 89-bp segment from the *psmα* operon, and found that the impact on PSMαs by depletion of *agrCA*, *agrD* or RNAIII were similar with that on PSMβs (**Figure 3**).

### The Functional Roles of PSMs in Agr Regulated Persister Formation

Based on the observation of different impacts on persister formation by mutation of AgrCA and RNAIII, we hypothesized that the gene(s) are regulated differently by AgrCA and RNAIII. It has been reported that AgrCA strongly up-regulates while RNAIII down-regulates expression of PSMαs and PSMβs in post-exponential phase (Queck et al., 2008). Here we showed the similar trends with regulation of *psm* genes by Agr CA and RNAIII in stationary phase, where AgrCA up regulated and RNAIII down regulated expression of both *psmα1-4* and *psm*β*1-2* (**Figures 2**, **3**).

**FIGURE 4:**
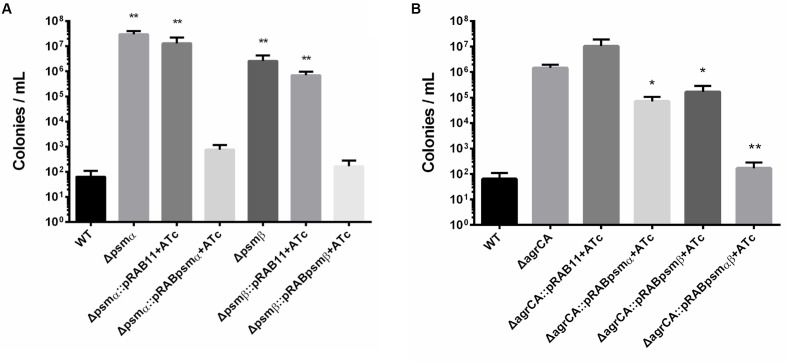
Phenol soluble modulins affect *S. aureus* persister formation. **(A,B)** Stationary phase USA500, knockout strains, complementary strains (based on pRB473) and overexpression strains (based on pRAB11) were treated with levofloxacin for 5 days. Results are representative of three independent experiments. ^∗^*p* < 0.05, ^∗∗^*p* < 0.01; *t*-tests vs. corresponding control sample (Δ*agrCA*).

We further constructed knockout strains *psmα1-4* or *psm*β*1-2*, which did not affect cell growth (**Supplementary Figure S2**). Persister assay showed that for stationary cultures, knockout of *psmα1-4* or *psm*β*1-2* resulted in significant enhancement of persister formation, similar to the effects of *agrCA* knockout. In addition, complementation of *psmα1-4* or *psm*β*1-2* reversed promotion of persister formation of each mutant, respectively (**Figure 4A**).

To investigate whether PSMs are the key factors in Agr regulated persister formation, we constructed overexpressing plasmids for *psmα1-4*, *psm*β*1-2* or both of them co-transcribed in one operon. Expressions of PSM genes are induced by ATc and not affected by absence of AgrA. Overexpression of *psmα1-4*, *psm*β*1-2* or both genes were capable of complementing the mutation of AgrCA on stationary phase persister formation, and the co-expressed PSMαs and PSMβs showed the strongest effects (**Figure 4B**). These results indicate that PSMαs and PSMβs are key downstream factors for persister regulation by the Agr system.

### NanK Is Involved in Persister Formation and L-Form Formation

**FIGURE 5:**
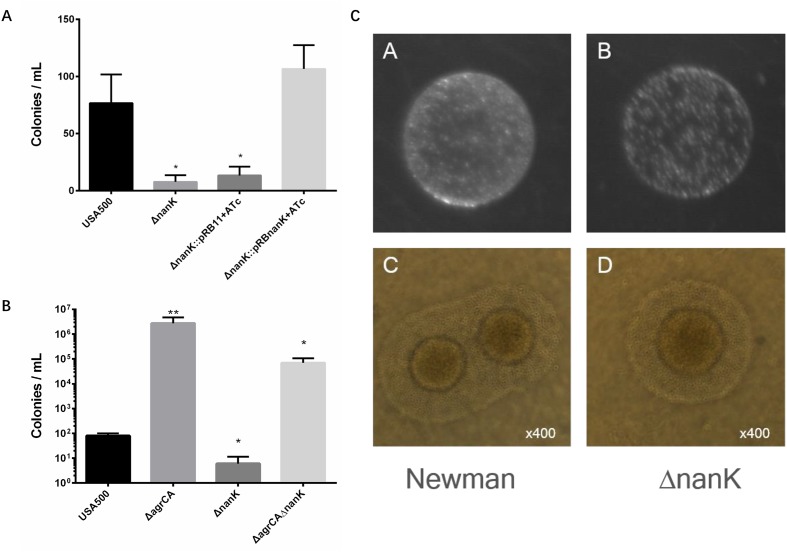
Comparison of persister formation and L-form formation of *S. aureus* strains. **(A,B)** Stationary phase USA500, knockout strains, complementary strains (based on pRB473) and double mutants were treated with levofloxacin. Results are representative of three independent experiments. ^∗^*p* < 0.05, ^∗∗^*p* < 0.01; *t* tests vs. corresponding control (WT) samples. **(C)** *S. aureus* USA500 strain and its *nanK* mutant were plated on LIM. (C,D) L-form colonies observed under microscope at 400× magnification. The typical “fried egg” morphology can be observed from both strains.

Persister formation is closely related to dormancy with low metabolic state. To find more genes responsible for Agr-mediated persister formation, we picked eight more metabolism associated genes (*hutU*, *narG*, *arcA*, *arcR*, *mtlD*, *imrP*, *rhbC*, and *nanK*) (**Table 2**) according to results of RNA-seq experiments performed by Queck et al. (2008) and also by us. Among these genes only *nanK* mutant showed decreased persister formation to levofloxacin in stationary phase, compared with USA500 strain while knockout of *nanK* did not affect bacterial growth (**Supplementary Figure S3**). In addition, the effects could be reversed by introduction of complementary plasmid pRBnanK (**Figure 5A**).

Since *nanK* expression is strongly up regulated in USA500 Δ*agrCA* strain which showed enhanced persister formation, we seek to detect whether knockout of *nanK* could affect persister formation of USA500 Δ*agrCA*. However, the double mutant showed similar persister level with that of USA500ΔagrCA (**Figure 5B**).

Sialic acid metabolism is an important bioavailable energy source in stationary phase (Olson et al., 2013). The Neu5Ac catabolic pathway is a complex process catalyzing *N*-acetylneuraminic acid (Neu5Ac) into Fructose-6P (Fru-6P), which can be utilized in central metabolism or cell wall metabolism. NanK, the ManNAc kinase, phosphorylates ManNAc at the C-6 position to yield ManNAc-6P plays an important role in the pathway (Almagro-Moreno and Boyd, 2009; Olson et al., 2013). To assess whether mutations of NanK or Agr system alter persister formation by affecting synthesis of ATP, intracellular ATP levels of stationary phase cultures of USA500 and its mutant strains of *nanK*, *agrCA*, *agrD*, *RNAIII*, *psmα1-4*, and *psm*β*1-2* were measured. However, no significant difference in ATP levels between these strains was found (**Supplementary Figure S4**).

We have found in our previous work that the glycerol uptake facilitator GlpF participates in both persister and L-form formation, indicating a close relationship between the two strategies bacteria use to survive challenge of antibiotics (Han et al., 2014). Having found that NanK deletion attenuated persister formation, we sought to investigate whether NanK is also involved in L-form formation. As shown in **Figure 5C**, Δ*nanK* formed fewer L-form colonies, while the shape and size of individual bacterial cells were similar with that of the wild type strain.

## Discussion

*Staphylococcus aureus* persister mechanisms are different from those in *E. coli* persisters in several aspects. In *E. coli*, the influence of mutation of a gene on persister formation is generally consistent, with variations on growth phases or type of antibiotics. However, *S. aureus* persister genes often affect persister formation only in exponential phase or stationary phase (Han et al., 2014; Xu et al., 2016). Also, the difference in persister formation can sometimes be observed when treated with specific antibiotics (Mechler et al., 2015; Wenjie et al., 2015; Yee et al., 2015). We also showed in this study that the impacts on persister formation by AgrCA and Agr regulated genes were only found from treatment with levofloxacin. However, the underlying mechanisms for the difference between *S. aureus* and *E. coli* persister formation are far from being unveiled and require further study.

Agr is one of the master regulators of virulence in *S. aureus.* Mutations of Agr system genes have been shown to strongly attenuate virulence (George and Muir, 2007; Cheung et al., 2011; Bünter et al., 2016). Based on its importance, several studies have focused on developing anti-Agr strategies in *S. aureus*, and obvious repression of pathogenicity has been confirmed (Gray et al., 2013; Murray et al., 2014; Khan et al., 2015). However, though anti-Agr strategies seem promising, many groups have reported that Agr dysfunction promotes persistent bacteremia (Fowler et al., 2004; Park et al., 2013; Kang et al., 2017), permits abscess formation (Das et al., 2016), and increases mortality among patients with *S. aureus* bacteremia, especially the most severe cases (Schweizer et al., 2011). The unexpected correlation of Agr dysfunction and worsened outcome of infection is intriguing and the reason remains unclear (Painter et al., 2014).

Previous studies showed that selection for loss of Agr-defective strains was mainly by healthcare environment including antibiotic treatment (Butterfield et al., 2011; Smyth et al., 2012). A possible explanation is that, for unknown reasons, Agr dysfunction decreases vancomycin bactericidal activity (Rose et al., 2007; Tsuji et al., 2009). However, the impacts of Agr dysfunction on MIC of vancomycin were about two to four fold. Meanwhile, we did not observe MIC change of the *agrCA* mutant treated with vancomycin or any other antibiotics used in this study (**Table 3**). Another hypothesis is that Agr dysfunction might help *S. aureus* escape host immune attack by reducing expression of multiple virulence genes regulated by Agr system (Malachowa et al., 2011). However, it is also reported that Agr dysfunction also promotes neutrophil lysis after phagocytosis (Surewaard et al., 2013). Therefore, the eventual outcome of interaction of host immune system and Agr dysfunction is yet to be determined.

**Table 3:** Minimum inhibitory concentration (MIC) (μg/μl) of *Staphylococcus aureus* strains used in this study.

Strains	Gentamicin	Levofloxacin	Oxacillin	Vancomycin
USA500	2	0.5	0.2	0.5
USA500ΔagrCA	2	0.5	0.2	0.5
USA500ΔagrD	2	0.5	0.2	0.5
USA500ΔRNAIII	1	0.5	0.2	0.5
USA500Δpsmα	2	0.5	0.2	0.5
USA500Δpsmβ	2	0.5	0.2	0.5
Newman	1	0.25	0.05	0.5
NewmanΔagrCA	1	0.25	0.05	0.5
HG003	1	0.25	0.1	0.5
HG003ΔagrCA	1	0.25	0.1	0.5

Based on our study, we provide another possible explanation that Agr dysfunction might promote persistent infection by increasing persister formation through down-regulation of PSMαs and PSMβs in *S. aureus*. This is demonstrated by elevated persister formation of Agr mutants in our study. The multifunctional PSMs have been reported to help *S. aureus* cope with immune system by killing human neutrophils (Wang et al., 2007). PSMs also mediate maturation and detachment of late phase staphylococcal biofilms (Otto, 2013), which significantly promotes staphylococcal persister formation (Shapiro et al., 2011; Conlon et al., 2015). Besides extracellular effects, PSMs also act as an intracellular signal to regulate gene expression. So far, PSMs have been found to bind only PmtR, which controls expression of genes in the *pmt* operon (Joo et al., 2016). More research is required to investigate whether PSMαs and PSMβs affect persister formation through PmtR, and if so, how PmtR regulate persister formation by controlling its target genes. Besides *in vitro* studies, more studies are required to address the regulation of persister formation by Agr system through PSMs in animal models, where the host immune system plays a role or additional antibiotic treatment may be applied to interrogate the role of Agr and PSM in persistent infection. Nonetheless, based on the clinical reports and our findings, we argue that the application of anti-Agr therapy should be more cautious especially when treating patients with persistent *S. aureus* infections, in particular for those with immunodeficiency.

We wondered what are the key factors in Agr mediated persister regulation. Agr system regulates different aspects of *S. aureus* biologic processes in both RNAIII dependent and RNA independent pathways. It is worth noticing that the study by Queck et al. (2008) and our research showed that Agr and RNAIII regulate some genes including *psmα1-4* and *psm*β*1-2* in opposite ways, in both exponential phase and stationary phase, respectively. In addition, a recent study showed that δ-toxin regulates colony spreading by inhibiting PSM binding to *S. aureus* surface (Kizaki et al., 2016). The role of RNAIII/δ-toxin in PSM regulated persister formation is worth further research.

Entry into stationary phase has been reported to promote expression of genes involved in sialic catabolism (Olson et al., 2013). Among the metabolism associated genes we studied by knockout and persister assay, only knockout of *nanK*, which was down-regulated by AgrCA and RNAIII (**Figure 2**), attenuated persister formation and L-form formation (**Figure 5**). It has been reported that ATP depletion is a key reason for persister increase in stationary phase (Conlon et al., 2016). However, no significant difference in ATP levels between Δ*nanK* and its parent strain was found (**Supplementary Figure S4**), indicating the existence of other persister mechanisms independent of ATP depletion as being involved in Agr mediated persister formation in *S. aureu*s. In addition, since sialic acid is more abundant in mucosa of hosts than in TSB (Mayer et al., 1964), the role of the sialic acid metabolism plays in Agr mediated persister formation might be more important *in vivo* and is worth further investigation in animal models.

L-form is an intriguing phenomenon and its mechanisms are not well unveiled in *S. aureus* (Owens and Nickerson, 1989; Michailova et al., 2007). Since L-form formation is closely related to cell wall integrity, a likely reason for NanK’s role in L-form formation is that NanK deletion decreased the production of Fru-6-P, which is a substrate for synthesis of UDP-GlcNAc, a crucial molecule for cell wall building. Combined with our previous findings with GlpF (Han et al., 2014), our finding that NanK deletion attenuated both persister formation and L-form formation indicates the close relationship between formation of L-forms and persisters (Zhang, 2014).

**FIGURE 6:**
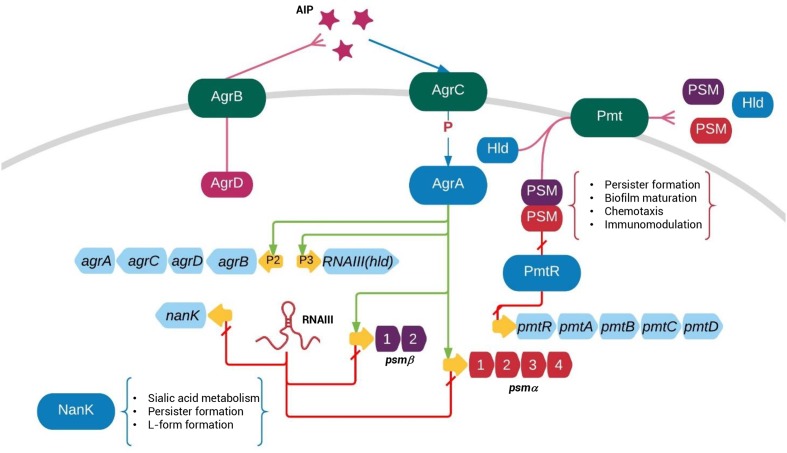
Regulation of persister formation is part of the Agr quorum-sensing regulatory network. The *agr* operon encodes a signal molecule AIP from P2 promoter and a regulatory RNA molecule RNAIII from P3 promoter. While AgrA up-regulates expression of P2, P3, NanK, and PSM expression, RNAIII negatively regulate NanK and PSM expression. NanK is involved in sialic metabolism and persister formation. PSMαs and PSMβs represses persister formation through unclear mechanisms. The “P” between AgrC and AgrA indicates phosphorylation.

In summary, by screening persister associated genes we showed that Agr system plays an important role in persister formation as seen by elevated persister levels in Agr mutants, in addition to its role in controlling virulence factors (**Figure 6**). The regulation of persister formation by Agr system is mainly carried out by PSMs. We also investigated the regulatory role of Agr system in sialic acid metabolism, which participates in persister formation and L-form formation. Our findings provide new insights into the biology of *S. aureus* persister formation and provides a possible explanation for the worsened bacteremia by Agr dysfunction in clinical treatments.

## Author Contributions

YZ, W-HZ, and TX designed the work and revised the manuscript. TX, X-YW, Y-MZ, and PC completed all the experiments. TX performed the statistically analysis, made the figures and wrote the manuscript.

## Conflict of Interest Statement

The authors declare that the research was conducted in the absence of any commercial or financial relationships that could be construed as a potential conflict of interest.

## Supplementary Material

FIGURE S1 **(A–D)** Exponential phase culture of USA500 and knockout mutants were treated with levofloxacin, oxacillin, gentamicin or vancomycin. **(E)** Persister levels of stationary phase cultures of USA500 and mutants. Results are representative of three independent experiments.

FIGURE S2 Growth curve of USA500 and the two knockout mutants of *psmα1-4* and *psm*β*1-2*.

FIGURE S3 Growth curve of USA500 and the its knockout mutants of *nanK*.

FIGURE S4 Intracellular concentration of ATP in stationary phase USA500 and mutant strains. The values were adjusted according to the CFU count of each sample.

## Abbreviations

MIC: minimum inhibitory concentration
MRSA: methicillin resistant *Staphylococcus aureus;*
MSSA: methicillin susceptible *Staphylococcus aureus*
PSM: phenol soluble modulins
QS: quorum sensing
TCS: two-component signal transduction system.

## References

[B1] Almagro-Moreno S. Boyd E. F. (2009). Insights into the evolution of sialic acid catabolism among bacteria. *BMC Evol. Biol.* 9:118. 10.1186/1471-2148-9-118 19470179 PMC2693436

[B2] Andrews J. M. (2001). Determination of minimum inhibitory concentrations. *J. Antimicrob. Chemother.* 48(Suppl. 1), 5–16. 10.1093/jac/48.suppl_1.5 11420333

[B3] Atshan S. S. Shamsudin M. N. Lung L. T. Ling K. H. Sekawi Z. Pei C. P. (2012). Improved method for the isolation of RNA from bacteria refractory to disruption, including *S. aureus* producing biofilm. *Gene* 494 219–224. 10.1016/j.gene.2011.12.010 22222139

[B4] Baba T. Bae T. Schneewind O. Takeuchi F. Hiramatsu K. (2008). Genome sequence of *Staphylococcus aureus* strain Newman and comparative analysis of staphylococcal genomes: polymorphism and evolution of two major pathogenicity islands. *J. Bacteriol.* 190 300–310. 10.1128/JB.01000-07 17951380 PMC2223734

[B5] Bae T. Schneewind O. (2006). Allelic replacement in *Staphylococcus aureus* with inducible counter-selection. *Plasmid* 55 58–63. 10.1016/j.plasmid.2005.05.005 16051359

[B6] Bigger J. (1944). Treatment of staphylococcal infections with penicillin by intermittent sterilisation. *Lancet* 244 497–500. 10.1016/S0140-6736(00)74210-3

[B7] Bruckner R. Wagner E. Gotz F. (1993). Characterization of a sucrase gene from *Staphylococcus xylosus*. *J. Bacteriol.* 175 851–857. 10.1128/jb.175.3.851-857.1993 8423155 PMC196229

[B8] Bünter J. P. Seth-Smith H. M. B. Rüegg S. Heikinheimo A. Borel N. Johler S. (2016). Wild type agr-negative livestock-associated MRSA exhibits high adhesive capacity to human and porcine cells. *Res. Microbiol.* 168 130–138. 10.1016/j.resmic.2016.09.006 27720828

[B9] Butterfield J. M. Tsuji B. T. Brown J. Ashley E. D. Hardy D. Brown K. (2011). Predictors of agr dysfunction in methicillin-resistant *Staphylococcus aureus* (MRSA) isolates among patients with MRSA bloodstream infections. *Antimicrob. Agents Chemother.* 55 5433–5437. 10.1128/AAC.00407-11 21930887 PMC3232784

[B10] Chatterjee S. S. Joo H. S. Duong A. C. Dieringer T. D. Tan V. Y. Song Y. (2013). Essential *Staphylococcus aureus* toxin export system. *Nat. Med.* 19 364–367. 10.1038/nm.3047 23396209 PMC3594369

[B11] Cheung G. Y. Wang R. Khan B. A. Sturdevant D. E. Otto M. (2011). Role of the accessory gene regulator agr in community-associated methicillin-resistant *Staphylococcus aureus* pathogenesis. *Infect. Immun.* 79 1927–1935. 10.1128/IAI.00046-11 21402769 PMC3088142

[B12] Cheverton A. M. Gollan B. Przydacz M. Wong C. T. Mylona A. Hare S. A. (2016). A *Salmonella* toxin promotes persister formation through acetylation of tRNA. *Mol. Cell.* 63 86–96. 10.1016/j.molcel.2016.05.002 27264868 PMC4942678

[B13] Cohen N. R. Lobritz M. A. Collins J. J. (2013). Microbial persistence and the road to drug resistance. *Cell Host Microbe* 13 632–642. 10.1016/j.chom.2013.05.009 23768488 PMC3695397

[B14] Conlon B. P. Nakayasu E. S. Fleck L. E. Lafleur M. D. Isabella V. M. Coleman K. (2013). Activated ClpP kills persisters and eradicates a chronic biofilm infection. *Nature* 503 365–370. 10.1038/nature12790 24226776 PMC4031760

[B15] Conlon B. P. Rowe S. E. Gandt A. B. Nuxoll A. S. Donegan N. P. Zalis E. A. (2016). Persister formation in *Staphylococcus aureus* is associated with ATP depletion. *Nat. Microbiol.* 1:16051. 10.1038/nmicrobiol.2016.51 27572649 PMC4932909

[B16] Conlon B. P. Rowe S. E. Lewis K. (2015). “Persister cells in biofilm associated infections,” in *Biofilm-Based Healthcare-Associated Infections*, Vol. II, ed. Donelli G. (Cham: Springer International Publishing), 1–9. 10.1007/978-3-319-09782-4_1 25384659

[B17] Corrigan R. M. Bellows L. E. Wood A. Gründling A. (2016). ppGpp negatively impacts ribosome assembly affecting growth and antimicrobial tolerance in Gram-positive bacteria. *Proc. Natl. Acad. Sci. U.S.A.* 113 E1710–E1719. 10.1073/pnas.1522179113 26951678 PMC4812758

[B18] Das S. Lindemann C. Young B. C. Muller J. Osterreich B. Ternette N. (2016). Natural mutations in a *Staphylococcus aureus* virulence regulator attenuate cytotoxicity but permit bacteremia and abscess formation. *Proc. Natl. Acad. Sci. U.S.A.* 113 E3101–E3110. 10.1073/pnas.1520255113 27185949 PMC4896717

[B19] Dhar N. McKinney J. D. (2007). Microbial phenotypic heterogeneity and antibiotic tolerance. *Curr. Opin. Microbiol.* 10 30–38. 10.1016/j.mib.2006.12.007 17215163

[B20] Diep B. A. Carleton H. A. Chang R. F. Sensabaugh G. F. Perdreau-Remington F. (2006). Roles of 34 virulence genes in the evolution of hospital- and community-associated strains of methicillin-resistant *Staphylococcus aureus*. *J. Infect. Dis.* 193 1495–1503. 10.1086/503777 16652276

[B21] Dorr T. Lewis K. Vulic M. (2009). SOS response induces persistence to fluoroquinolones in *Escherichia coli*. *PLOS Genet.* 5:e1000760. 10.1371/journal.pgen.1000760 20011100 PMC2780357

[B22] Dunman P. M. Murphy E. Haney S. Palacios D. Tucker-Kellogg G. Wu S. (2001). Transcription profiling-based identification of *Staphylococcus aureus* genes regulated by the agr and/or sarA loci. *J. Bacteriol.* 183 7341–7353. 10.1128/JB.183.24.7341-7353.2001 11717293 PMC95583

[B23] Fauvart M. De Groote V. N. Michiels J. (2011). Role of persister cells in chronic infections: clinical relevance and perspectives on anti-persister therapies. *J. Med. Microbiol.* 60(Pt 6), 699–709. 10.1099/jmm.0.0309320 21459912

[B24] Fowler V. G. Sakoulas G. McIntyre L. M. Meka V. G. Arbeit R. D. Cabell C. H. (2004). Persistent bacteremia due to methicillin-resistant *Staphylococcus aureus* infection is associated with agr dysfunction and low-level in vitro resistance to thrombin-induced platelet microbicidal protein. *J. Infect. Dis.* 190 1140–1149. 10.1086/423145 15319865

[B25] Geiger T. Goerke C. Fritz M. Schafer T. Ohlsen K. Liebeke M. (2010). Role of the (p)ppGpp synthase RSH, a RelA/SpoT homolog, in stringent response and virulence of *Staphylococcus aureus*. *Infect. Immun.* 78 1873–1883. 10.1128/iai.01439-09 20212088 PMC2863498

[B26] George E. A. Muir T. W. (2007). Molecular mechanisms of agr quorum sensing in virulent staphylococci. *Chembiochem* 8 847–855. 10.1002/cbic.200700023 17457814

[B27] Germain E. Roghanian M. Gerdes K. Maisonneuve E. (2015). Stochastic induction of persister cells by HipA through (p)ppGpp-mediated activation of mRNA endonucleases. *Proc. Natl. Acad. Sci. U.S.A.* 112 5171–5176. 10.1073/pnas.1423536112 25848049 PMC4413331

[B28] Gray B. Hall P. Gresham H. (2013). Targeting agr- and agr-Like quorum sensing systems for development of common therapeutics to treat multiple gram-positive bacterial infections. *Sensors* 13 5130–5166. 10.3390/s130405130 23598501 PMC3673130

[B29] Han J. He L. Shi W. Xu X. Wang S. Zhang S. (2014). Glycerol uptake is important for L-form formation and persistence in *Staphylococcus aureus*. *PLOS ONE* 9:e108325. 10.1371/journal.pone.0108325 25251561 PMC4177120

[B30] Helle L. Kull M. Mayer S. Marincola G. Zelder M. E. Goerke C. (2011). Vectors for improved Tet repressor-dependent gradual gene induction or silencing in *Staphylococcus aureus*. *Microbiology* 157(Pt 12), 3314–3323. 10.1099/mic.0.052548-0 21921101

[B31] Herbert S. Ziebandt A. K. Ohlsen K. Schafer T. Hecker M. Albrecht D. (2010). Repair of global regulators in *Staphylococcus aureus* 8325 and comparative analysis with other clinical isolates. *Infect. Immun.* 78 2877–2889. 10.1128/IAI.00088-10 20212089 PMC2876537

[B32] Hobby G. L. Meyer K. Chaffee E. (1942). Observations on the mechanism of action of penicillin. *Exp. Biol. Med.* 50 281–285. 10.3181/00379727-50-13773

[B33] Ji G. Beavis R. C. Novick R. P. (1995). Cell density control of staphylococcal virulence mediated by an octapeptide pheromone. *Proc. Natl. Acad. Sci. U.S.A.* 92 12055–12059. 10.1073/pnas.92.26.12055 8618843 PMC40295

[B34] Joo H.-S. Chatterjee S. S. Villaruz A. E. Dickey S. W. Tan V. Y. Chen Y. (2016). Mechanism of gene regulation by a *Staphylococcus aureus* toxin. *mBio* 7:e01579-16. 10.1128/mBio.01579-16 27795396 PMC5080381

[B35] Kang C. K. Kim Y. K. Jung S. I. Park W. B. Song K. H. Park K. H. (2017). agr functionality affects clinical outcomes in patients with persistent methicillin-resistant *Staphylococcus aureus* bacteraemia. *Eur. J. Clin. Microbiol. Infect. Dis.* 36 2187–2191. 10.1007/s10096-017-3044-2 28639163

[B36] Khan B. A. Yeh A. J. Cheung G. Y. C. Otto M. (2015). Investigational therapies targeting quorum-sensing for the treatment of *Staphylococcus aureus* infections. *Expert Opin. Investig. Drugs* 24 689–704. 10.1517/13543784.2015.1019062 25704585 PMC6106785

[B37] Kim Y. Wang X. Ma Q. Zhang X. S. Wood T. K. (2009). Toxin-antitoxin systems in *Escherichia coli* influence biofilm formation through YjgK (TabA) and fimbriae. *J. Bacteriol.* 191 1258–1267. 10.1128/JB.01465-08 19060153 PMC2632003

[B38] Kizaki H. Omae Y. Tabuchi F. Saito Y. Sekimizu K. Kaito C. (2016). Cell-surface phenol soluble modulins regulate *Staphylococcus aureus* colony spreading. *PLOS ONE* 11:e0164523. 10.1371/journal.pone.0164523 27723838 PMC5056675

[B39] Korch S. B. Henderson T. A. Hill T. M. (2003). Characterization of the hipA7 allele of *Escherichia coli* and evidence that high persistence is governed by (p)ppGpp synthesis. *Mol. Microbiol.* 50 1199–1213. 10.1046/j.1365-2958.2003.03779.x 14622409

[B40] Lechner S. Lewis K. Bertram R. (2012). *Staphylococcus aureus* persisters tolerant to bactericidal antibiotics. *J. Mol. Microbiol. Biotechnol.* 22 235–244. 10.1159/000342449 22986269 PMC3518770

[B41] Leung V. Lévesque C. M. (2012). A stress-inducible quorum-sensing peptide mediates the formation of persister cells with noninherited multidrug tolerance. *J. Bacteriol.* 194 2265–2274. 10.1128/jb.06707-11 22366415 PMC3347057

[B42] Lewis K. (2007). Persister cells, dormancy and infectious disease. *Nat. Rev. Microbiol.* 5 48–56. 10.1038/nrmicro1557 17143318

[B43] Li J. Ji L. Shi W. Xie J. Zhang Y. (2013). Trans-translation mediates tolerance to multiple antibiotics and stresses in *Escherichia coli*. *J. Antimicrob. Chemother.* 68 2477–2481. 10.1093/jac/dkt231 23812681 PMC3797643

[B44] Lina G. Jarraud S. Ji G. Greenland T. Pedraza A. Etienne J. (1998). Transmembrane topology and histidine protein kinase activity of AgrC, the agr signal receptor in *Staphylococcus aureus*. *Mol. Microbiol.* 28 655–662. 10.1046/j.1365-2958.1998.00830.x 9632266

[B45] Livak K. J. Schmittgen T. D. (2001). Analysis of relative gene expression data using real-time quantitative PCR and the 2^-ΔΔC_t_^ Method. *Methods* 25 402–408. 10.1006/meth.2001.1262 11846609

[B46] Ma C. Sim S. Shi W. Du L. Xing D. Zhang Y. (2010). Energy production genes sucB and ubiF are involved in persister survival and tolerance to multiple antibiotics and stresses in *Escherichia coli*. *FEMS Microbiol. Lett.* 303 33–40. 10.1111/j.1574-6968.2009.01857.x 20041955

[B47] Maisonneuve E. Castro-Camargo M. Gerdes K. (2013). (p)ppGpp controls bacterial persistence by stochastic induction of toxin-antitoxin activity. *Cell* 154 1140–1150. 10.1016/j.cell.2013.07.048 23993101

[B48] Malachowa N. Whitney A. R. Kobayashi S. D. Sturdevant D. E. Kennedy A. D. Braughton K. R. (2011). Global changes in *Staphylococcus aureus* gene expression in human blood. *PLOS ONE* 6:e18617. 10.1371/journal.pone.0018617 21525981 PMC3078114

[B49] Mayer F. C. Dam R. Pazur J. H. (1964). Occurence of sialic acids in plant seeds. *Arch. Biochem. Biophys.* 108 356–357. 10.1016/0003-9861(64)90398-4 14240590

[B50] Mechler L. Herbig A. Paprotka K. Fraunholz M. Nieselt K. Bertram R. (2015). A novel point mutation promotes growth phase-dependent daptomycin tolerance in *Staphylococcus aureus*. *Antimicrob. Agents Chemother.* 59 5366–5376. 10.1128/AAC.00643-15 26100694 PMC4538524

[B51] Michailova L. Kussovsky V. Radoucheva T. Jordanova M. Markova N. (2007). Persistence of *Staphylococcus aureus* L-form during experimental lung infection in rats. *FEMS Microbiol. Lett.* 268 88–97. 10.1111/j.1574-6968.2006.00567.x 17168999

[B52] Mina E. G. Marques C. N. (2016). Interaction of *Staphylococcus aureus* persister cells with the host when in a persister state and following awakening. *Sci. Rep.* 6:31342. 10.1038/srep31342 27506163 PMC4979210

[B53] Mitchell A. M. Wang W. Silhavy T. J. (2016). Novel RpoS-dependent mechanisms strengthen the envelope permeability barrier during stationary phase. *J. Bacteriol.* 199:e00708-16. 10.1128/JB.00708-16 27821607 PMC5198486

[B54] Moker N. Dean C. R. Tao J. (2010). *Pseudomonas aeruginosa* increases formation of multidrug-tolerant persister cells in response to quorum-sensing signaling molecules. *J. Bacteriol.* 192 1946–1955. 10.1128/jb.01231-09 20097861 PMC2838031

[B55] Monk I. R. Shah I. M. Xu M. Tan M. W. Foster T. J. (2012). Transforming the untransformable: application of direct transformation to manipulate genetically *Staphylococcus aureus* and *Staphylococcus epidermidis*. *mBio* 3:e00277-11. 10.1128/mBio.00277-11 22434850 PMC3312211

[B56] Morfeldt E. Taylor D. von Gabain A. Arvidson S. (1995). Activation of alpha-toxin translation in *Staphylococcus aureus* by the trans-encoded antisense RNA, RNAIII. *EMBO J.* 14 4569–4577. 7556100 10.1002/j.1460-2075.1995.tb00136.x PMC394549

[B57] Mulcahy L. R. Burns J. L. Lory S. Lewis K. (2010). Emergence of *Pseudomonas aeruginosa* strains producing high levels of persister cells in patients with cystic fibrosis. *J. Bacteriol.* 192 6191–6199. 10.1128/JB.01651-09 20935098 PMC2981199

[B58] Murray E. J. Crowley R. C. Truman A. Clarke S. R. Cottam J. A. Jadhav G. P. (2014). Targeting *Staphylococcus aureus* quorum sensing with nonpeptidic small molecule inhibitors. *J. Med. Chem.* 57 2813–2819. 10.1021/jm500215s 24592914 PMC4010551

[B59] Olson M. E. King J. M. Yahr T. L. Horswill A. R. (2013). Sialic acid catabolism in *Staphylococcus aureus*. *J. Bacteriol.* 195 1779–1788. 10.1128/jb.02294-12 23396916 PMC3624546

[B60] Otto M. (2013). Staphylococcal infections: mechanisms of biofilm maturation and detachment as critical determinants of pathogenicity. *Annu. Rev. Med.* 64 175–188. 10.1146/annurev-med-042711-140023 22906361

[B61] Owens W. E. Nickerson S. C. (1989). Morphologic study of *Staphylococcus aureus* L-form, reverting, and intermediate colonies in situ. *J. Clin. Microbiol.* 27 1382–1386. 2754006 10.1128/jcm.27.6.1382-1386.1989 PMC267563

[B62] Painter K. L. Krishna A. Wigneshweraraj S. Edwards A. M. (2014). What role does the quorum-sensing accessory gene regulator system play during *Staphylococcus aureus* bacteremia? *Trends Microbiol.* 22 676–685. 10.1016/j.tim.2014.09.002 25300477

[B63] Park S. Y. Chong Y. P. Park H. J. Park K. H. Moon S. M. Jeong J. Y. (2013). agr Dysfunction and persistent methicillin-resistant *Staphylococcus aureus* bacteremia in patients with removed eradicable foci. *Infection* 41 111–119. 10.1007/s15010-012-0348-0 23065454

[B64] Peschel A. Otto M. (2013). Phenol-soluble modulins and staphylococcal infection. *Nat. Rev. Microbiol.* 11 667–673. 10.1038/nrmicro3110 24018382 PMC4780437

[B65] Pu Y. Zhao Z. Li Y. Zou J. Ma Q. Zhao Y. (2016). Enhanced efflux activity facilitates drug tolerance in dormant bacterial cells. *Mol. Cell.* 62 284–294. 10.1016/j.molcel.2016.03.035 27105118 PMC4850422

[B66] Queck S. Y. Jameson-Lee M. Villaruz A. E. Bach T. H. Khan B. A. Sturdevant D. E. (2008). RNAIII-independent target gene control by the agr quorum-sensing system: insight into the evolution of virulence regulation in *Staphylococcus aureus*. *Mol. Cell.* 32 150–158. 10.1016/j.molcel.2008.08.005 18851841 PMC2575650

[B67] Rose W. E. Rybak M. J. Tsuji B. T. Kaatz G. W. Sakoulas G. (2007). Correlation of vancomycin and daptomycin susceptibility in *Staphylococcus aureus* in reference to accessory gene regulator (agr) polymorphism and function. *J. Antimicrob. Chemother.* 59 1190–1193. 10.1093/jac/dkm091 17434881

[B68] Schumacher M. A. Balani P. Min J. Chinnam N. B. Hansen S. Vulic M. (2015). HipBA-promoter structures reveal the basis of heritable multidrug tolerance. *Nature* 524 59–64. 10.1038/nature14662 26222023 PMC7502270

[B69] Schweizer M. L. Furuno J. P. Sakoulas G. Johnson J. K. Harris A. D. Shardell M. D. (2011). Increased mortality with accessory gene regulator (agr) dysfunction in *Staphylococcus aureus* among bacteremic patients. *Antimicrob. Agents Chemother.* 55 1082–1087. 10.1128/aac.00918-10 21173172 PMC3067101

[B70] Shan Y. Brown Gandt A. Rowe S. E. Deisinger J. P. Conlon B. P. Lewis K. (2017). ATP-dependent persister formation in *Escherichia coli*. *mBio* 8:e02267-16. 10.1128/mBio.02267-16 28174313 PMC5296605

[B71] Shapiro J. A. Nguyen V. L. Chamberlain N. R. (2011). Evidence for persisters in *Staphylococcus epidermidis* RP62a planktonic cultures and biofilms. *J. Med. Microbiol.* 60(Pt 7), 950–960. 10.1099/jmm.0.026013-0 21415203

[B72] Singh R. Ray P. Das A. Sharma M. (2009). Role of persisters and small-colony variants in antibiotic resistance of planktonic and biofilm-associated *Staphylococcus aureus*: an in vitro study. *J. Med. Microbiol.* 58(Pt 8), 1067–1073. 10.1099/jmm.0.009720-0 19528167

[B73] Smyth D. S. Kafer J. M. Wasserman G. A. Velickovic L. Mathema B. Holzman R. S. (2012). Nasal carriage as a source of agr-defective *Staphylococcus aureus* bacteremia. *J. Infect. Dis.* 206 1168–1177. 10.1093/infdis/jis483 22859823 PMC3448967

[B74] Surewaard B. G. J. de Haas C. J. C. Vervoort F. Rigby K. M. DeLeo F. R. Otto M. (2013). Staphylococcal alpha-phenol soluble modulins contribute to neutrophil lysis after phagocytosis. *Cell Microbiol.* 15 1427–1437. 10.1111/cmi.12130 23470014 PMC4784422

[B75] Tsuji B. T. Harigaya Y. Lesse A. J. Sakoulas G. Mylotte J. M. (2009). Loss of vancomycin bactericidal activity against accessory gene regulator (agr) dysfunctional *Staphylococcus aureus* under conditions of high bacterial density. *Diagn. Microbiol. Infect. Dis.* 64 220–224. 10.1016/j.diagmicrobio.2009.01.028 19345040

[B76] Wang R. Braughton K. R. Kretschmer D. Bach T. H. Queck S. Y. Li M. (2007). Identification of novel cytolytic peptides as key virulence determinants for community-associated MRSA. *Nat. Med.* 13 1510–1514. 10.1038/nm1656 17994102

[B77] Wenjie W. Jiazhen C. Gang C. Xin D. Peng C. Jing W. (2015). Transposon mutagenesis identifies novel genes associated with *Staphylococcus aureus* persister formation. *Front. Microbiol.* 6:1437. 10.3389/fmicb.2015.01437 26779120 PMC4689057

[B78] World Health Organization (2017). *Global Priority List of Antibiotic-Resistant Bacteria to Guide Research, Discovery, and Development of New Antibiotics.* Available at: http://www.who.int/medicines/publications/global-priority-list-antibiotic-resistant-bacteria/en/

[B79] Xu T. Han J. Zhang J. Chen J. Wu N. Zhang W. (2016). Absence of protoheme IX Farnesyltransferase CtaB causes virulence attenuation but enhances pigment production and persister survival in MRSA. *Front. Microbiol.* 7:1625. 10.3389/fmicb.2016.01625 27822202 PMC5076432

[B80] Yee R. Cui P. Shi W. Feng J. Zhang Y. (2015). Genetic screen reveals the role of purine metabolism in *Staphylococcus aureus* persistence to Rifampicin. *Antibiotics* 4 627–642. 10.3390/antibiotics4040627 27025643 PMC4790316

[B81] Zhang Y. (2014). Persisters, persistent infections and the Yin–Yang model. *Emerg. Microb. Infect.* 3 e3. 10.1038/emi.2014.3 26038493 PMC3913823

[B82] Zhang Y. Yew W. W. Barer M. R. (2012). Targeting persisters for tuberculosis control. *Antimicrob. Agents Chemother.* 56 2223–2230. 10.1128/aac.06288-11 22391538 PMC3346619

[B83] Ziebandt A. K. Becher D. Ohlsen K. Hacker J. Hecker M. Engelmann S. (2004). The influence of agr and sigmaB in growth phase dependent regulation of virulence factors in *Staphylococcus aureus*. *Proteomics* 4 3034–3047. 10.1002/pmic.200400937 15378746

