# Supplementary figures and images for: The Agr Quorum Sensing System Represses Persister Formation through Regulation of Phenol Soluble Modulins in Staphylococcus aureus
Source: Front Microbiol. 2017 Nov 7;8:2189. doi: 10.3389/fmicb.2017.02189 (PMC5681930; doi:10.3389/fmicb.2017.02189)

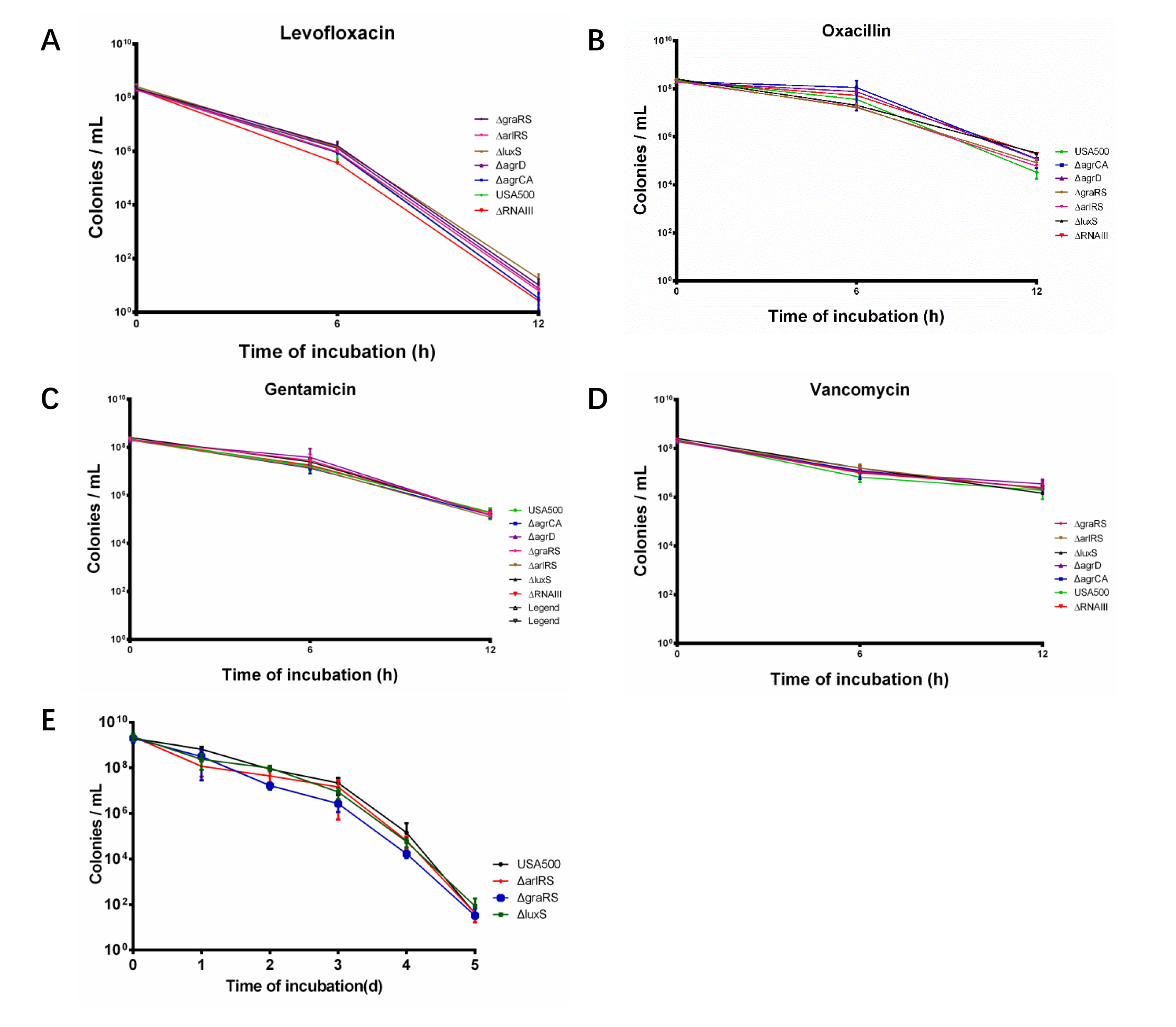

Supplement: FIGURE S1 — (A–D) Exponential phase culture of USA500 and knockout mutants were treated with levofloxacin, oxacillin, gentamicin or vancomycin. (E) Persister levels of stationary phase cultures of USA500 and mutants. Results are representative of three independent experiments. [file Image_1.TIF]

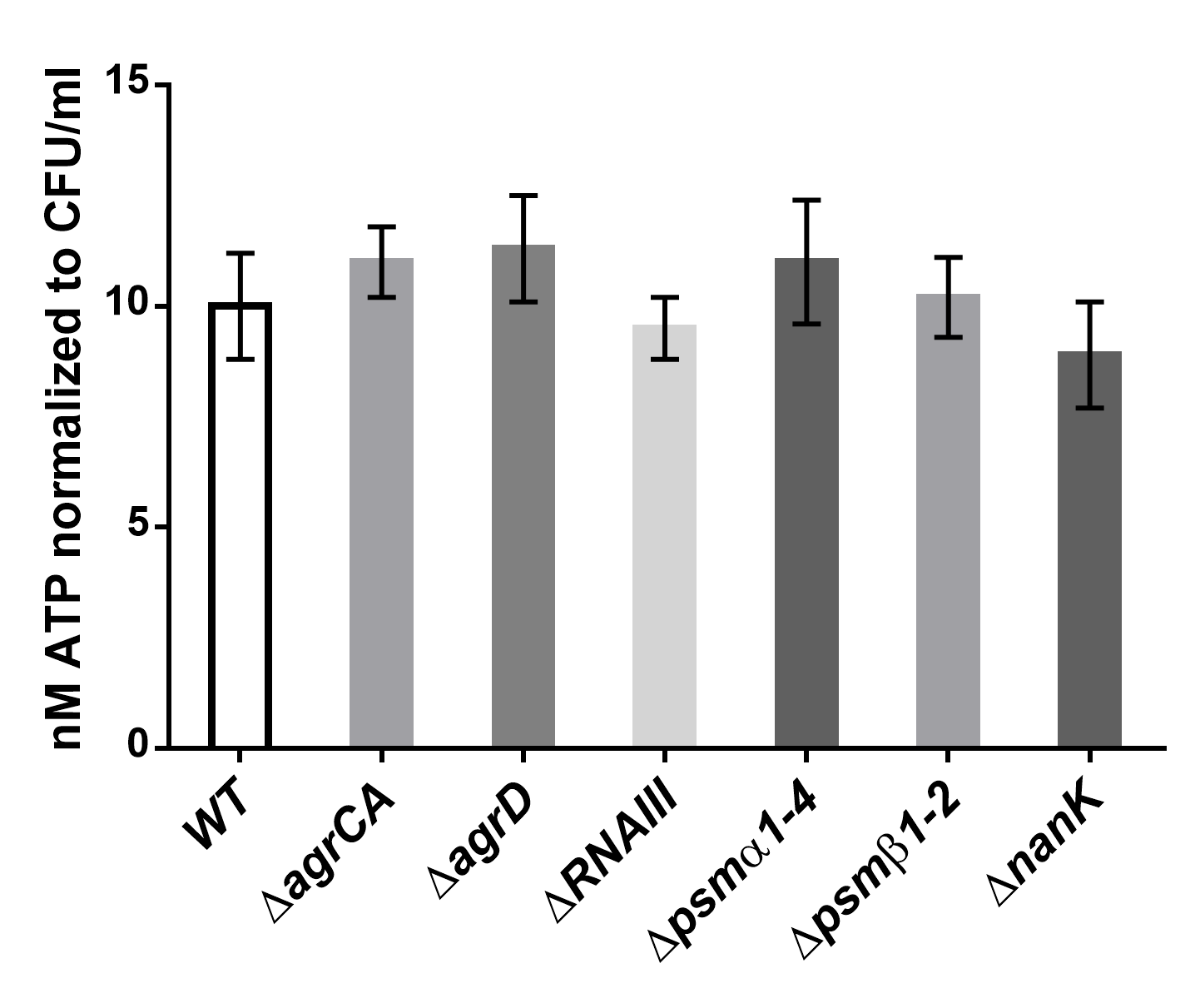

Supplement: FIGURE S4 — Intracellular concentration of ATP in stationary phase USA500 and mutant strains. The values were adjusted according to the CFU count of each sample. [file Image_4.TIF]
