# Supplementary material for: The Agr Quorum Sensing System Represses Persister Formation through Regulation of Phenol Soluble Modulins in Staphylococcus aureus
Source: Front Microbiol. 2017 Nov 7;8:2189. doi: 10.3389/fmicb.2017.02189 (PMC5681930; doi:10.3389/fmicb.2017.02189)
Supplement: Supplementary file 5 [file Data_Sheet_1.docx]

**Supplementary Table 1. Primers used in this study.**

| **Primer** | **Sequence (5’→3’)** |
| --- | --- |
| **For construction of knockout strains** | |
| agrCA-uf-KpnI | GGGGTACCCACTGTGTCGATAATCCATTTTAC |
| agrCA-ur | GCCGTTAACTGACTTTATTATCTTACTACTATCACACTCTCTATTTAAATTATTC |
| agrCA-df | GAATAATTTAAATAGAGAGTGTGATAGTAGTAAGATAATAAAGTCAGTTAACGGC |
| agrCA-dr-EcoRI | CGGAATTCACAGTTCAAAAGTTAGGACAAC |
| agrD-uf-KpnI | GGGGTACCTCACTTGCTAATTTTACATGGTTATTAAG |
| agrD-ur | CACCTACTATCACACTCTCTATTTAAATCATTTTAAGTCCTCCTTAATAAAGAAA |
| agrD-df | TTTCTTTATTAAGGAGGACTTAAAATGATTTAAATAGAGAGTGTGATAGTAGGTG |
| agrD-dr-MluI | CGACGCGTCAATAAATGCAACGCGAATGATAG |
| RNAIII-uf-KpnI | GGGGTACCTTTAAATGAAAAAGCAGATAATAATCTAGGAC |
| RNAIII-ur | GGAAAATAAATGCTTTTAGCATGTTTTAATAAGAGTTAGTTTCCTTGGACTC |
| RNAIII-df | GAGTCCAAGGAAACTAACTCTTATTAAAACATGCTAAAAGCATTTATTTTCC |
| RNAIII-dr-MluI | CGACGCGTCGACGCGTGCTGGTATTGTAAACATTAATATCATTTGA |
| psmα-uf-KpnI | GGGGTACCAGTCGCAATATAAATAGTTACAATTGC |
| psmα-ur | AGAATTTTAAGTATTCAATTCGCTTAAATAAGATTACCTCCTTTGCTTATG |
| psmα-df | CATAAGCAAAGGAGGTAATCTTATTTAAGCGAATTGAATACTTAAAATTCT |
| psmα-dr-EcoRI | CGGAATTCATTAAGACAACAAATTCTGAAGTAGATTT |
| psmβ-uf-KpnI | GGGGTACCTATATCGAAACAAACAGGAGTTC |
| psmβ-ur | CCCAGTTTATTTTAAAGAATATTAGTTATTATATGAAAACACTCCTTAAAATTTAAATT |
| psmβ-df | AATTTAAATTTTAAGGAGTGTTTTCATATAATAACTAATATTCTTTAAAATAAACTGGG |
| psmβ-dr-Eco52I | cccggccGGAGCTAGAATGGCATTACT |
| luxS-uf-KpnI | GGGGTACCATTCATAATCACAACCAGTCTGT |
| luxS-ur | CTTTTCTGATTAACTTGATTGACTAAGATTTTGAATTTCCTCCTATTAACTACTC |
| luxS-df | GAGTAGTTAATAGGAGGAAATTCAAAATCTTAGTCAATCAAGTTAATCAGAAAAG |
| luxS-dr-EcoRI | CGGAATTCATTAGAAGATGCTGAAGCATTAG |
| arlRS-uf-KpnI | GGGGTACCAAAAAACAAAAGCAGTAAACCTAAAG |
| arlRS-ur | GACGTCTCAGTCATGACCTCATATTACGACTTTTTCTAA |
| arlRS-df | TTAGAAAAAGTCGTAATATGAGGTCATGACTGAGACGTC |
| arlRS-dr-MluI | CGACGCGTGCATTGTACCGTATGAATCAG |
| graRS-uf-KpnI | GGGGTACCGTTTACTAGCCGATGTGACG |
| graRS-ur | ATCAATTCTAAGTAACAAAACGCATGTCCATATCACCCAATATCATTTAGTATATTT |
| graRS-df | AAATATACTAAATGATATTGGGTGATATGGACATGCGTTTTGTTACTTAGAATTGAT |
| graRS-dr-MluI | CGACGCGTGACTTGTGAGCCTTCCTTTAT |
| nanK-uf-KpnI | GGGGTACCTTTTGCAATGAGTTGATCTAATGTTTG |
| nanK-ur | AATGACTCCTTCTTTCAACTACTTACTCACTCCATTTCTATTG |
| nanK-df | CAATAGAAATGGAGTGAGTAAGTAGTTGAAAGAAGGAGTCATT |
| nanK-dr-MluI | CGACGCGTCCATAAGTCGTTTTTTCACGTAAAG |
| hutU-uf-KpnI | GGGGTACCGTCATTAATTTTTATACGATGATCTGCG |
| hutU-ur | CTGCTATATGATTAATTATTAAATCATTCATAAAATTCGCTCCTGTTCTTTTAAG |
| hutU-df | CTTAAAAGAACAGGAGCGAATTTTATGAATGATTTAATAATTAATCATATAGCAG |
| hutU-dr-MluI | CGACGCGTCCTTACCGTCTTTAATAACTTTTTCTG |
| narG-uf-KpnI | GGGGTACCGCGTCGATATAACAAGGTTG |
| narG-ur | AACTTGCGCTTTAATCTTCAAATTTATATCCTCCTACGTATAAAAATAC |
| narG-df | GTATTTTTATACGTAGGAGGATATAAATTTGAAGATTAAAGCGCAAGTT |
| narG-dr-MluI | CGACGCGTATCTCCTGCAGTGAAAATATTAGC |
| arcA-uf-KpnI | GGGGTACCCTCGGCAGTCTAAAATCATTAC |
| arcA-ur | ACCCTCGTTTCTATAATTTATTACGGTCTATTTCCTCCTTTTATCTTTG |
| arcA-df | CAAAGATAAAAGGAGGAAATAGACCGTAATAAATTATAGAAACGAGGGT |
| arcA-dr-Eco52I | CCCGGCCGCATTTCTCCTTTAATACCAATATGAGG |
| arcR-uf-KpnI | GGGGTACCGTTACTTTATATTCCAGCTCTTCTTGTTT |
| arcR-ur | GTTCAACTTATTCATTTTACATTGTATAAAGTTAAACTCCTTCAAACCTTG |
| arcR-df | CAAGGTTTGAAGGAGTTTAACTTTATACAATGTAAAATGAATAAGTTGAAC |
| arcR-dr-Eco52I | TTGCGGCCGCGACGCGTCCATAAATGTGTTACCACTTTGATTAG |
| mtlD-uf-KpnI | GGGGTACCTGTTCATGTTGAAACGAAAGATG |
| mtlD-ur | CTTGTTTCAAACAAAGCTCAATAAATTATGCATCTGCCTCCT |
| mtlD-df | AGGAGGCAGATGCATAATTTATTGAGCTTTGTTTGAAACAAG |
| mtlD-dr-MluI | CGACGCGTTGTAACAGAAACTGATAATGCAACG |
| imrP-uf-KpnI | GGGGTACCTATGGATTTATTAATACTTCTTAGAAACTATGAAG |
| imrP-ur | GATGCATAAAATAAATATTTAAATAAATTCACATTAATAACTACACCCCTTTC |
| imrP-df | GAAAGGGGTGTAGTTATTAATGTGAATTTATTTAAATATTTATTTTATGCATC |
| imrP-dr-MluI | CGACGCGTCATTATATGATTAGCTAAATACACACTAAC |
| rhbC-uf-KpnI | GGGGTACGTACGTGCCCATTGACTAAAT |
| rhbC-ur | CTCACGATTAATAAAATTCTATAACCTTACATAATTCACCTCTATGAAATATTTTAC |
| rhbC-df | GTAAAATATTTCATAGAGGTGAATTATGTAAGGTTATAGAATTTTATTAATCGTGAG |
| rhbC-dr-MluI | CGACGCGTCGGAATAATCATCAAATTTATTTTTGAACTTC |
| ipdC-uf-KpnI | GGGGTACCGAATTACCATATGCAGTATTAGAAGC |
| ipdC-ur | CTTATACCCTGTATACGATGAAATTACATAAAAAAAGCCACCTTTTCTC |
| ipdC-df | GAGAAAAGGTGGCTTTTTTTATGTAATTTCATCGTATACAGGGTATAAG |
| ipdC-dr-MluI | CGACGCGTGATAACTAGATATAAGGCAATCGTGA |
| **For construction of complementary expression (CE) or overexpression (OE) plasmids** | |
| CEagrCA-uf-KpnI | CGGGATCCTGTTAAAATATTAAATACAAATTACATTTAACAG |
| CEagrCA-ur | AAACAAAATTATAACTATTTAATAATTCCACTTTTACACCACTCTCCTCA |
| CEagrCA-df | TGAGGAGAGTGGTGTAAAAGTGGAATTATTAAATAGTTATAATTTTGTTT |
| CEagrCA-dr-EcoRI | CGGAATTCTTATATTTTTTTAACGTTTCTCACCGATG |
| CEagrD-uf-BamHI | CGGGATCCTGTTAAAATATTAAATACAAATTACATTTAACAG |
| CEagrD-ur | AATCAAAAAATAAGTTAAATAATGTATTCATTTTTACACCACTCTCCTCA |
| CEagrD-df | TGAGGAGAGTGGTGTAAAAATGAATACATTATTTAACTTATTTTTTGATT |
| CEagrD-dr-EcoRI | CGGAATTCCTATTTAAATTATTCGTGTAATTGTGTTAATTC |
| CErecA-f-BamHI | CGGGATCCGCACGTTTGTTCGTTTTTGCGTT |
| CErecA-r-EcoRI | CGGAATTCAGTTTAACTATAGATATAAATTT |
| CEnanK-f-BamHI | CGGGATCCTATGAAGTCCCTCCAAAAATC |
| CEnanK-r-EcoRI | CGGAATTCTAGAATGACTCCTTCTTTCAACTA |
| OEpsmα-f-KpnI | GGGGTACCAAAGGAGGTAATCTTAATGGGTATC |
| OEpsmα-r-BglII | GAAGATCTTATTGGTATAGTGGCCTGAGAAT |
| OEpsmβ-f-KpnI | GGGGTACCAATTTTAAGGAGTGTTTTCAATGG |
| OEpsmβ-r-BglII | GAAGATCTAAGTATGCTCACCCAGTTTATTTT |
| OEpsmαβ-ur | CCATTGAAAACACTCCTTAAAATTTTATTTTGCGAAAATGTCGATAATT |
| OEpsmαβ-df | AATTATCGACATTTTCGCAAAATAAAATTTTAAGGAGTGTTTTCAATGG |
| **For qRT-PCR** | |
| RTpsmα-f | TATCATCGCTGGCATCATTA |
| RTpsmα-r | AGACCTCCTTTGTTTGTTATG |
| RTpsmβ1-f | CAAAGGTGAGGGAGAGATT |
| RTpsmβ1-r | TTAGCAACGATGTCTACGA |
| RTcap5A-f | CGCAACTTATCAACATCCA |
| RTcap5A-r | GCACCGATTAGATTCACTAC |
| RTnanK-f | ATCACTGTCTCCTTATGTCAA |
| RTnanK-r | CCTGTACCAAGCGTCATA |
