# Supplementary material for: The Agr Quorum Sensing System Represses Persister Formation through Regulation of Phenol Soluble Modulins in Staphylococcus aureus
Source: Front Microbiol. 2017 Nov 7;8:2189. doi: 10.3389/fmicb.2017.02189 (PMC5681930; doi:10.3389/fmicb.2017.02189)
Supplement: Supplementary file 6 [file Data_Sheet_2.docx]

**Supplementary Table 2. RNA-seq differently expressed genes (ΔagrCA/USA500)**

| **Gene id** | **Gene name** | **Description** | **log2FC** | ***p* value** |
| --- | --- | --- | --- | --- |
| USA300HOU_RS10955 | *hld* | delta_hemolysin | -11.16 | 0.00E+00 |
| USA300HOU_RS05900 | *psmβ2* | hypothetical_protein | -10.99 | 0.00E+00 |
| USA300HOU_RS05895 | *psmβ1* | hypothetical_protein | -9.93 | 0.00E+00 |
| USA300HOU_RS10965 | *agrD* | hypothetical_protein | -7.19 | 4.56E-113 |
| USA300HOU_RS10960 | *agrB* | accessory_gene_regulator_protein_B | -5.89 | 0.00E+00 |
| USA300HOU_RS14500 | *lip* | lipase | -3.87 | 0.00E+00 |
| USA300HOU_RS01700 | *lip1* | lipase | -2.75 | 0.00E+00 |
| USA300HOU_RS03960 | *USA300HOU_0757* | hypothetical_protein | -2.44 | 1.32E-02 |
| USA300HOU_RS07225 | *USA300HOU_1351* | hypothetical_protein | -2.18 | 7.80E-03 |
| USA300HOU_RS10580 | *-* | hypothetical_protein | -2.18 | 3.91E-02 |
| USA300HOU_RS00405 | *-* | hypothetical_protein | -2.11 | 1.88E-05 |
| USA300HOU_RS08085 | *USA300HOU_1514* | hypothetical_protein | -2.07 | 3.07E-05 |
| USA300HOU_RS01005 | *USA300HOU_0204* | hypothetical_protein | -2.05 | 8.53E-178 |
| murQ | *USA300HOU_0205* | N_acetylmuramic_acid_6_phosphate_etherase | -1.95 | 2.07E-138 |
| USA300HOU_RS08530 | *USA300HOU_1602* | membrane_protein | -1.86 | 6.93E-03 |
| USA300HOU_RS10075 | *rrl3* | - | -1.67 | 3.16E-02 |
| USA300HOU_RS00800 | *cap5A* | capsular_polysaccharide_type_5_biosynthesis_protein_cap5A | -1.60 | 1.52E-02 |
| USA300HOU_RS09635 | *splE* | serine_protease_SplE | -1.59 | 3.94E-05 |
| USA300HOU_RS01015 | *USA300HOU_0206* | permease | -1.58 | 8.04E-112 |
| USA300HOU_RS03480 | *USA300HOU_0663* | antibiotic_ABC_transporter_ATP_binding_protein | -1.49 | 4.87E-113 |
| USA300HOU_RS01020 | *USA300HOU_0207* | RpiR_family_transcriptional_regulator | -1.49 | 6.06E-93 |
| USA300HOU_RS09630 | *splF* | serine_protease_SplF | -1.47 | 6.80E-05 |
| USA300HOU_RS00655 | *USA300HOU_0136* | hypothetical_protein | -1.44 | 1.13E-02 |
| USA300HOU_RS03335 | *USA300HOU_0634* | hypothetical_protein | -1.39 | 9.62E-03 |
| USA300HOU_RS03125 | *USA300HOU_0592* | hypothetical_protein | -1.37 | 1.84E-04 |
| USA300HOU_RS05145 | *-* | hypothetical_protein | -1.35 | 4.21E-09 |
| USA300HOU_RS10490 | *pmtR* | membrane_protein | -1.33 | 4.17E-08 |
| USA300HOU_RS10710 | *USA300HOU_1978* | hypothetical_protein | -1.30 | 3.00E-02 |
| USA300HOU_RS09505 | *USA300HOU_1779* | transposase | -1.24 | 1.76E-02 |
| USA300HOU_RS00815 | *-* | hypothetical_protein | -1.23 | 8.02E-05 |
| USA300HOU_RS09645 | *splC* | serine_protease_SplC | -1.16 | 6.86E-04 |
| USA300HOU_RS10500 | *pmtB* | GntR_family_transcriptional_regulator | -1.07 | 8.01E-33 |
| USA300HOU_RS05890 | *USA300HOU_t0016* | - | -1.01 | 1.32E-02 |
| USA300HOU_RS00300 | *USA300HOU_0065* | transposase | -1.00 | 3.35E-03 |
|  |  |  | -1.00 |  |
| USA300HOU_RS06055 | *pyrE* | orotate_phosphoribosyltransferase | 1.01 | 9.68E-20 |
| USA300HOU_RS09410 | *-* | transcriptional_regulator | 1.02 | 1.06E-04 |
| USA300HOU_RS04280 | *emp* | hypothetical_protein | 1.05 | 9.53E-19 |
| USA300HOU_RS05775 | *USA300HOU_1090* | fibrinogen_binding_protein | 1.11 | 1.67E-40 |
| USA300HOU_RS06050 | *pyrF* | orotidine_5_phosphate_decarboxylase | 1.12 | 5.33E-27 |
| USA300HOU_RS13740 | *-* | membrane_protein | 1.12 | 7.62E-30 |
| USA300HOU_RS01460 | *USA300HOU_0292* | membrane_protein | 1.13 | 2.85E-05 |
| USA300HOU_RS02525 | *USA300HOU_0494* | hypothetical_protein | 1.13 | 6.47E-46 |
| USA300HOU_RS05885 | *USA300HOU_1111* | DNA_binding_protein | 1.14 | 2.92E-03 |
| USA300HOU_RS14180 | *betT* | choline_transporter_BetT | 1.15 | 1.20E-40 |
| USA300HOU_RS06040 | *carA* | carbamoyl_phosphate_synthase_small_chain | 1.19 | 9.78E-37 |
| USA300HOU_RS00900 | *USA300HOU_0183* | hypothetical_protein | 1.20 | 7.35E-08 |
| USA300HOU_RS01695 | *USA300HOU_0339* | histidine_transporter | 1.22 | 3.12E-09 |
| USA300HOU_RS06045 | *carB* | carbamoyl_phosphate_synthase_large_chain | 1.23 | 1.69E-59 |
| USA300HOU_RS13925 | *USA300HOU_2561* | CHAP_domain_containing_protein | 1.26 | 1.54E-15 |
| USA300HOU_RS05130 | *-* | hypothetical_protein | 1.27 | 5.13E-03 |
| USA300HOU_RS05865 | *arcC2* | carbamate_kinase_1 | 1.31 | 2.82E-70 |
| USA300HOU_RS05850 | *USA300HOU_1104* | hypothetical_protein | 1.60 | 9.37E-04 |
| USA300HOU_RS01665 | *USA300HOU_0333* | pyrimidine_nucleoside_transporter_NupC | 1.69 | 2.07E-48 |
| USA300HOU_RS05855 | *USA300HOU_1105* | hypothetical_protein | 1.70 | 1.53E-06 |
| USA300HOU_RS09435 | *rpoE1* | RNA_polymerase_sigma_factor_SigS | 1.73 | 2.80E-03 |
| USA300HOU_RS05860 | *arcB2* | ornithine_carbamoyltransferase | 1.75 | 2.98E-118 |
| USA300HOU_RS01660 | *USA300HOU_0332* | pseudouridine_5_phosphate_glycosidase | 1.95 | 2.50E-54 |
| USA300HOU_RS01655 | *USA300HOU_0331* | carbohydrate_kinase | 2.24 | 3.97E-60 |
| USA300HOU_RS08220 | *USA300HOU_1541* | competence_protein_ComGF | 2.46 | 3.91E-02 |
| USA300HOU_RS00185 | *nanK* | ManNAc kinase | 3.31 | 4.27E-04 |
