# Supplementary material for: The Agr Quorum Sensing System Represses Persister Formation through Regulation of Phenol Soluble Modulins in Staphylococcus aureus
Source: Front Microbiol. 2017 Nov 7;8:2189. doi: 10.3389/fmicb.2017.02189 (PMC5681930; doi:10.3389/fmicb.2017.02189)
Supplement: Supplementary file 7 [file Data_Sheet_3.docx]

**Supplementary Table 3. RNA-seq differently expressed genes (ΔagrD/USA500)**

| **Gene id** | **Gene name** | **Description** | **log2FC** | **Pvalue** |
| --- | --- | --- | --- | --- |
| USA300HOU_RS10955 | *hld* | delta_hemolysin | -11.96 | 0.0E+00 |
| USA300HOU_RS05895 | *psmβ1* | hypothetical_protein | -9.77 | 0.0E+00 |
| USA300HOU_RS05900 | *psmβ2* | hypothetical_protein | -9.09 | 0.0E+00 |
| USA300HOU_RS10975 | *agrA* | DNA_binding_response_regulator | -6.04 | 0.0E+00 |
| USA300HOU_RS10970 | *agrC* | histidine_kinase | -6.00 | 0.0E+00 |
| USA300HOU_RS10960 | *agrB* | accessory_gene_regulator_protein_B | -5.98 | 0.0E+00 |
| USA300HOU_RS14500 | *lip* | lipase | -4.00 | 0.0E+00 |
| USA300HOU_RS07325 | *USA300HOU_1370* | hypothetical_protein | -3.07 | 3.9E-02 |
| USA300HOU_RS08530 | *USA300HOU_1602* | membrane_protein | -2.81 | 5.2E-04 |
| USA300HOU_RS01700 | *lip1* | lipase | -2.62 | 3.7E-305 |
| USA300HOU_RS03335 | *USA300HOU_0634* | hypothetical_protein | -2.45 | 2.1E-04 |
| USA300HOU_RS01005 | *USA300HOU_0204* | hypothetical_protein | -2.40 | 4.7E-228 |
| murQ | *USA300HOU_0205* | N_acetylmuramic_acid_6_phosphate_etherase | -2.39 | 1.4E-176 |
| USA300HOU_RS01295 | *USA300HOU_0260* | hypothetical_protein | -2.39 | 3.9E-02 |
| USA300HOU_RS00175 | *USA300HOU_0034* | transposase | -2.32 | 2.7E-03 |
| USA300HOU_RS13135 | *bioD* | ATP_dependent_dethiobiotin_synthetase | -2.27 | 9.9E-04 |
| USA300HOU_RS13065 | *USA300HOU_2397* | hypothetical_protein | -2.21 | 2.8E-06 |
| USA300HOU_RS01015 | *USA300HOU_0206* | permease | -2.08 | 9.7E-174 |
| USA300HOU_RS01955 | *-* | integrase | -2.07 | 1.2E-02 |
| USA300HOU_RS01020 | *USA300HOU_0207* | RpiR_family_transcriptional_regulator | -2.02 | 6.9E-154 |
| USA300HOU_RS11740 | *USA300HOU_2159* | multidrug_resistance_efflux_pump_SepA | -1.92 | 4.2E-10 |
| USA300HOU_RS13845 | *USA300HOU_2545* | ferrous_iron_transporter_A | -1.88 | 6.3E-03 |
| USA300HOU_RS02010 | *USA300HOU_0402* | hypothetical_protein | -1.74 | 7.1E-09 |
| USA300HOU_RS00405 | *-* | hypothetical_protein | -1.73 | 2.4E-04 |
| USA300HOU_RS11930 | *USA300HOU_2195* | MerR_family_transcriptional_regulator | -1.66 | 1.9E-04 |
| USA300HOU_RS03150 | *USA300HOU_0597* | hypothetical_protein | -1.66 | 3.2E-04 |
| USA300HOU_RS03025 | *USA300HOU_0571* | hypothetical_protein | -1.63 | 6.3E-06 |
| USA300HOU_RS14495 | *icaC* | poly_beta_1_2C6_N_acetyl_D_glucosamine_export_protein | -1.60 | 4.3E-03 |
| USA300HOU_RS03480 | *USA300HOU_0663* | antibiotic_ABC_transporter_ATP_binding_protein | -1.58 | 2.2E-118 |
| USA300HOU_RS11070 | *ilvH* | acetolactate_synthase | -1.57 | 6.6E-05 |
| USA300HOU_RS05375 | *-* | hypothetical_protein | -1.56 | 1.1E-15 |
| USA300HOU_RS03610 | *sarX* | transcriptional_regulator | -1.53 | 4.0E-18 |
| USA300HOU_RS10480 | *USA300HOU_1933* | membrane_protein | -1.45 | 1.0E-11 |
| USA300HOU_RS10490 | *pmtR* | membrane_protein | -1.42 | 1.3E-08 |
| USA300HOU_RS02080 | *USA300HOU_0416* | mRNA_interferase_PemK | -1.39 | 1.8E-02 |
| USA300HOU_RS14645 | *USA300HOU_2702* | membrane_protein | -1.39 | 8.2E-72 |
| USA300HOU_RS04100 | *USA300HOU_0785* | hypothetical_protein | -1.33 | 4.5E-04 |
| USA300HOU_RS00655 | *USA300HOU_0136* | hypothetical_protein | -1.33 | 2.6E-02 |
| USA300HOU_RS14135 | *USA300HOU_2601* | membrane_protein | -1.32 | 1.5E-06 |
| USA300HOU_RS03400 | *mnhE1* | cation_proton_antiporter | -1.31 | 5.3E-16 |
| USA300HOU_RS00605 | *USA300HOU_0127* | siderophore_biosynthesis_protein_SbnA | -1.25 | 7.3E-03 |
| USA300HOU_RS12790 | *USA300HOU_2346* | LytTR_family_transcriptional_regulator | -1.23 | 1.3E-07 |
| USA300HOU_RS05145 | *-* | hypothetical_protein | -1.22 | 4.8E-08 |
| USA300HOU_RS12245 | *-* | membrane_protein | -1.22 | 3.0E-04 |
| USA300HOU_RS04000 | *USA300HOU_0766* | hypothetical_protein | -1.20 | 4.3E-02 |
| USA300HOU_RS06980 | *trpF* | phosphoribosylanthranilate_isomerase | -1.20 | 4.3E-02 |
| USA300HOU_RS01555 | *USA300HOU_0311* | membrane_protein | -1.17 | 2.6E-03 |
| USA300HOU_RS03140 | *USA300HOU_0595* | hypothetical_protein | -1.15 | 1.0E-02 |
| USA300HOU_RS04315 | *-* | hypothetical_protein | -1.12 | 2.9E-02 |
| USA300HOU_RS03275 | *sarA* | transcriptional_regulator | -1.12 | 8.0E-52 |
| USA300HOU_RS08645 | *USA300HOU_1624* | hypothetical_protein | -1.09 | 7.6E-43 |
| USA300HOU_RS11955 | *USA300HOU_2200* | toxin | -1.07 | 1.2E-48 |
| USA300HOU_RS13380 | *USA300HOU_2459* | hypothetical_protein | -1.07 | 4.9E-03 |
| USA300HOU_RS09505 | *USA300HOU_1779* | transposase | -1.07 | 4.0E-02 |
| USA300HOU_RS01610 | *USA300HOU_0322* | hypothetical_protein | -1.05 | 1.2E-06 |
| USA300HOU_RS06695 | *USA300HOU_1261* | hypothetical_protein | -1.05 | 1.0E-03 |
| USA300HOU_RS04505 | *USA300HOU_0868* | hypothetical_protein | -1.03 | 1.1E-54 |
| USA300HOU_RS01750 | *USA300HOU_0350* | PTS_ascorbate_transporter_subunit_IIC | -1.03 | 2.1E-38 |
| USA300HOU_RS10850 | *USA300HOU_2007* | hypothetical_protein | -1.02 | 3.2E-06 |
| USA300HOU_RS12625 | *fosB* | metallothiol_transferase_FosB | -1.02 | 4.6E-02 |
| USA300HOU_RS03125 | *USA300HOU_0592* | hypothetical_protein | -1.02 | 3.2E-03 |
| USA300HOU_RS10500 | *pmtB* | GntR_family_transcriptional_regulator | -1.01 | 1.9E-28 |
| USA300HOU_RS10530 | *hlb1* | phospholipase | -1.01 | 9.8E-05 |
| USA300HOU_RS12535 | *USA300HOU_2298* | RpiR_family_transcriptional_regulator | -1.00 | 1.4E-38 |
| USA300HOU_RS05775 | *USA300HOU_1090* | fibrinogen_binding_protein | 1.02 | 2.5E-38 |
| USA300HOU_RS07970 | *USA300HOU_1492* | hypothetical_protein | 1.05 | 1.0E-07 |
| USA300HOU_RS13925 | *USA300HOU_2561* | CHAP_domain_containing_protein | 1.12 | 9.5E-14 |
| USA300HOU_RS07065 | *pstC* | phosphate_ABC_transporter_permease | 1.14 | 3.9E-15 |
| USA300HOU_RS05865 | *arcC2* | carbamate_kinase_1 | 1.22 | 1.2E-66 |
| USA300HOU_RS06595 | *USA300HOU_1241* | XRE_family_transcriptional_regulator | 1.32 | 2.5E-02 |
| USA300HOU_RS01665 | *USA300HOU_0333* | pyrimidine_nucleoside_transporter_NupC | 1.36 | 6.9E-33 |
| USA300HOU_RS05860 | *arcB2* | ornithine_carbamoyltransferase | 1.59 | 1.0E-104 |
| USA300HOU_RS01660 | *USA300HOU_0332* | pseudouridine_5_phosphate_glycosidase | 1.72 | 2.1E-44 |
| USA300HOU_RS09365 | *USA300HOU_1754* | transporter | 1.77 | 2.3E-03 |
| USA300HOU_RS04855 | *USA300HOU_0938* | membrane_protein | 1.80 | 4.0E-03 |
| USA300HOU_RS01655 | *USA300HOU_0331* | carbohydrate_kinase | 1.89 | 3.5E-44 |
| USA300HOU_RS00185 | *nanK* | ManNAc kinase | 3.10 | 4.3E-04 |
| USA300HOU_RS10060 | *USA300HOU_t0049* | - | 3.25 | 1.2E-02 |
