# Supplementary material for: The Agr Quorum Sensing System Represses Persister Formation through Regulation of Phenol Soluble Modulins in Staphylococcus aureus
Source: Front Microbiol. 2017 Nov 7;8:2189. doi: 10.3389/fmicb.2017.02189 (PMC5681930; doi:10.3389/fmicb.2017.02189)
Supplement: Supplementary file 8 [file Data_Sheet_4.docx]

**Supplementary Table 4. RNA-seq differently expressed genes (ΔRNAIII/USA500)**

| **Gene id** | **Gene name** | **Description** | **log2FC** | **Pvalue** |
| --- | --- | --- | --- | --- |
| USA300HOU_RS14500 | *lip* | lipase | -3.57 | 0.0E+00 |
| USA300HOU_RS06680 | *USA300HOU_1257* | hypothetical_protein | -3.45 | 3.1E-05 |
| USA300HOU_RS10795 | *USA300HOU_1996* | hypothetical_protein | -3.15 | 1.2E-02 |
| USA300HOU_RS00800 | *cap5A* | capsular_polysaccharide_type_5_biosynthesis_protein_cap5A | -3.02 | 1.6E-03 |
| USA300HOU_RS13695 | *USA300HOU_2515* | peptide_ABC_transporter_ATP_binding_protein | -2.86 | 2.7E-03 |
| USA300HOU_RS09635 | *splE* | serine_protease_SplE | -2.61 | 1.3E-07 |
| USA300HOU_RS01700 | *lip1* | lipase | -2.61 | 3.2E-300 |
| USA300HOU_RS01005 | *USA300HOU_0204* | hypothetical_protein | -2.59 | 8.4E-250 |
| murQ | *USA300HOU_0205* | N_acetylmuramic_acid_6_phosphate_etherase | -2.59 | 6.8E-185 |
| USA300HOU_RS04845 | *leuA1* | 2_isopropylmalate_synthase | -2.28 | 1.2E-02 |
| USA300HOU_RS01470 | *USA300HOU_0294* | membrane_protein | -2.15 | 3.9E-02 |
| USA300HOU_RS01015 | *USA300HOU_0206* | permease | -2.09 | 6.3E-170 |
| USA300HOU_RS01020 | *USA300HOU_0207* | RpiR_family_transcriptional_regulator | -2.09 | 1.7E-154 |
| USA300HOU_RS14165 | *USA300HOU_2607* | hypothetical_protein | -2.02 | 3.2E-02 |
| USA300HOU_RS14240 | *-* | hypothetical_protein | -1.97 | 3.3E-04 |
| USA300HOU_RS08085 | *USA300HOU_1514* | hypothetical_protein | -1.90 | 4.5E-04 |
| USA300HOU_RS10555 | *chp* | chemotaxis_inhibitory_protein | -1.74 | 2.5E-06 |
| USA300HOU_RS03175 | *USA300HOU_0603* | hypothetical_protein | -1.69 | 8.6E-03 |
| USA300HOU_RS05830 | *USA300HOU_1100* | membrane_protein | -1.67 | 7.4E-05 |
| USA300HOU_RS13470 | *-* | hypothetical_protein | -1.66 | 1.9E-04 |
| USA300HOU_RS10255 | *USA300HOU_1887* | hypothetical_protein | -1.64 | 4.9E-03 |
| USA300HOU_RS14495 | *icaC* | poly_beta_1_2C6_N_acetyl_D_glucosamine_export_protein | -1.59 | 7.4E-03 |
| USA300HOU_RS03335 | *USA300HOU_0634* | hypothetical_protein | -1.59 | 7.4E-03 |
| USA300HOU_RS09505 | *USA300HOU_1779* | transposase | -1.59 | 7.4E-03 |
| USA300HOU_RS03355 | *USA300HOU_0638* | hypothetical_protein | -1.57 | 4.7E-03 |
| USA300HOU_RS13135 | *bioD* | ATP_dependent_dethiobiotin_synthetase | -1.41 | 2.5E-02 |
| USA300HOU_RS03480 | *USA300HOU_0663* | antibiotic_ABC_transporter_ATP_binding_protein | -1.39 | 2.2E-91 |
| USA300HOU_RS03315 | *USA300HOU_0630* | cell_division_protein_FtsK | -1.36 | 7.8E-04 |
| USA300HOU_RS00405 | *-* | hypothetical_protein | -1.36 | 4.2E-03 |
| USA300HOU_RS01560 | *USA300HOU_0312* | hypothetical_protein | -1.36 | 4.2E-03 |
| USA300HOU_RS01425 | *USA300HOU_0285* | hypothetical_protein | -1.34 | 1.2E-05 |
| USA300HOU_RS13360 | *USA300HOU_2455* | peptide_ABC_transporter_ATP_binding_protein | -1.23 | 1.0E-06 |
| USA300HOU_RS02035 | *-* | hypothetical_protein | -1.22 | 4.4E-02 |
| USA300HOU_RS04380 | *USA300HOU_0841* | hypothetical_protein | -1.22 | 4.4E-02 |
| USA300HOU_RS03970 | *USA300HOU_0759* | iron_ABC_transporter_permease | -1.21 | 1.0E-04 |
| USA300HOU_RS09645 | *splC* | serine_protease_SplC | -1.18 | 1.1E-03 |
| USA300HOU_RS03975 | *USA300HOU_0760* | iron_ABC_transporter_permease | -1.18 | 4.9E-02 |
| USA300HOU_RS10550 | *USA300HOU_1946* | hypothetical_protein | -1.17 | 6.9E-46 |
| USA300HOU_RS02205 | *USA300HOU_0440* | hypothetical_protein | -1.11 | 2.0E-03 |
| USA300HOU_RS02190 | *USA300HOU_0437* | hypothetical_protein | -1.10 | 3.7E-39 |
| USA300HOU_RS05785 | *USA300HOU_1092* | FPRL1_inhibitory_protein | -1.10 | 2.4E-05 |
| USA300HOU_RS09630 | *splF* | serine_protease_SplF | -1.08 | 4.0E-03 |
| USA300HOU_RS08670 | *USA300HOU_1627* | hypothetical_protein | -1.00 | 9.8E-03 |
| USA300HOU_RS10490 | *USA300HOU_1935* | membrane_protein | 1.02 | 6.3E-09 |
| USA300HOU_RS06740 | *USA300HOU_1270* | hypothetical_protein | 1.03 | 1.1E-15 |
| USA300HOU_RS07385 | *USA300HOU_1380* | hypothetical_protein | 1.03 | 8.2E-06 |
| USA300HOU_RS09830 | *USA300HOU_1832* | hypothetical_protein | 1.04 | 4.0E-02 |
| USA300HOU_RS01695 | *USA300HOU_0339* | histidine_transporter | 1.08 | 1.8E-07 |
| USA300HOU_RS13130 | *bioA* | adenosylmethionine_8_amino_7_oxononanoate_aminotransferase_BioA | 1.10 | 5.7E-04 |
| USA300HOU_RS03370 | *USA300HOU_0641* | hypothetical_protein | 1.11 | 3.7E-02 |
| USA300HOU_RS02080 | *USA300HOU_0416* | mRNA_interferase_PemK | 1.12 | 3.0E-03 |
| USA300HOU_RS12445 | *USA300HOU_2281* | hypothetical_protein | 1.13 | 5.8E-17 |
| USA300HOU_RS10485 | *USA300HOU_1934* | ABC_transporter_ATP_binding_protein | 1.13 | 4.7E-54 |
| USA300HOU_RS09945 | *USA300HOU_t0025* | - | 1.14 | 3.1E-03 |
| USA300HOU_RS10500 | *USA300HOU_1937* | GntR_family_transcriptional_regulator | 1.15 | 3.1E-48 |
| USA300HOU_RS12770 | *htrB* | hemin_ABC_transporter_ATP_binding_protein | 1.23 | 1.8E-71 |
| USA300HOU_RS05530 | *-* | membrane_protein | 1.25 | 6.0E-19 |
| USA300HOU_RS05600 | *USA300HOU_1059* | hypothetical_protein | 1.25 | 1.7E-05 |
| USA300HOU_RS10495 | *USA300HOU_1936* | ABC_transporter_ATP_binding_protein | 1.26 | 9.4E-66 |
| USA300HOU_RS08180 | *USA300HOU_1533* | membrane_protein | 1.27 | 1.1E-03 |
| USA300HOU_RS01665 | *USA300HOU_0333* | pyrimidine_nucleoside_transporter_NupC | 1.29 | 1.3E-27 |
| USA300HOU_RS02405 | *-* | hypothetical_protein | 1.31 | 4.2E-03 |
| USA300HOU_RS08245 | *USA300HOU_1546* | hypothetical_protein | 1.31 | 5.0E-03 |
| USA300HOU_RS01660 | *USA300HOU_0332* | pseudouridine_5_phosphate_glycosidase | 1.38 | 3.5E-25 |
| USA300HOU_RS11595 | *USA300HOU_2138* | hypothetical_protein | 1.48 | 1.3E-102 |
| USA300HOU_RS00940 | *USA300HOU_0191* | multidrug_MFS_transporter | 1.51 | 3.4E-86 |
| USA300HOU_RS05885 | *USA300HOU_1111* | DNA_binding_protein | 1.52 | 1.5E-05 |
| USA300HOU_RS06725 | *USA300HOU_1267* | membrane_protein | 1.53 | 4.2E-02 |
| USA300HOU_RS09435 | *rpoE1* | RNA_polymerase_sigma_factor_SigS | 1.53 | 1.1E-02 |
| USA300HOU_RS12775 | *htrA* | hemin_ABC_transporter_permease | 1.55 | 5.2E-107 |
| USA300HOU_RS01655 | *USA300HOU_0331* | carbohydrate_kinase | 1.59 | 2.1E-27 |
| USA300HOU_RS05115 | *-* | hypothetical_protein | 1.62 | 2.6E-70 |
| USA300HOU_RS10480 | *USA300HOU_1933* | membrane_protein | 1.74 | 8.7E-36 |
| USA300HOU_RS06595 | *USA300HOU_1241* | XRE_family_transcriptional_regulator | 1.89 | 1.4E-03 |
| USA300HOU_RS05900 | *psmβ1* | hypothetical_protein | 2.16 | 1.1E-214 |
| USA300HOU_RS05895 | *psmβ2* | hypothetical_protein | 2.49 | 4.4E-288 |
| USA300HOU_RS00185 | *nanK* | ManNAc kinase | 2.63 | 2.3E-02 |
